# Mathematical modelling of SigE regulatory network reveals new insights into bistability of mycobacterial stress response

**DOI:** 10.1186/s12859-021-04372-5

**Published:** 2021-11-19

**Authors:** Irene Zorzan, Simone Del Favero, Alberto Giaretta, Riccardo Manganelli, Barbara Di Camillo, Luca Schenato

**Affiliations:** 1 Department of Information Engineering, University of Padova, 35131 Padova, Italy; 2 Department of Molecular Medicine, University of Padova, 35121 Padova, Italy; 3 Department of Comparative Biomedicine and Food Science, University of Padova, 35020 Legnaro (Padova), Italy

**Keywords:** Bacterial persistence, Mathematical modelling, Bistability

## Abstract

**Background:**

The ability to rapidly adapt to adverse environmental conditions represents the key of success of many pathogens and, in particular, of *Mycobacterium tuberculosis*. Upon exposition to heat shock, antibiotics or other sources of stress, appropriate responses in terms of genes transcription and proteins activity are activated leading part of a genetically identical bacterial population to express a different phenotype, namely to develop persistence. When the stress response network is mathematically described by an ordinary differential equations model, development of persistence in the bacterial population is associated with bistability of the model, since different emerging phenotypes are represented by different stable steady states.

**Results:**

In this work, we develop a mathematical model of SigE stress response network that incorporates interactions not considered in mathematical models currently available in the literature. We provide, through involved analytical computations, accurate approximations of the system’s nullclines, and exploit the obtained expressions to determine, in a reliable though computationally efficient way, the number of equilibrium points of the system.

**Conclusions:**

Theoretical analysis and perturbation experiments point out the crucial role played by the degradation pathway involving RseA, the anti-sigma factor of SigE, for coexistence of two stable equilibria and the emergence of bistability. Our results also indicate that a fine control on RseA concentration is a necessary requirement in order for the system to exhibit bistability.

**Supplementary Information:**

The online version contains supplementary material available at 10.1186/s12859-021-04372-5.

## Background

*Mycobacterium tuberculosis*, like many other bacteria, can survive adverse environmental conditions thanks to its ability to sense environmental changes and start appropriate responses in genes expression and proteins activity. These responses allow a fraction of a clonal (i.e., genetically identical) bacterial population to survive exposure to stress and to persist for much longer periods of time with respect to the remaining (non-persistent) subpopulation. Expression of the persistent phenotype, a phenomenon known as “bacterial persistence” [1, 2], is the primary challenge in the fight against *Mycobacterium tuberculosis* as persistent cells escape elimination by the immune system [3] and can remain dormant for years before starting replication [4].

Generally speaking, bacteria achieve adaptation to stress conditions of diverse nature (e.g., antibiotics, surface or oxidative stress, heat shock) by activating specific groups of genes controlled by a variety of sigma factors whose number is associated with the environmental variability experienced by the bacterial species. Availability of sigma factors results from their competition for core RNA polymerase [5] and from modulation of their transcription, translation, proteolysis and sequestering by anti-sigma factors [6]. Sigma factors regulatory networks involve multiple elements (kinases, sigma and anti-sigma factors) which interact through intertwined positive and negative feedback loops. Disentangling the intricate set of interactions and unraveling the role played by each component (e.g., specific feedback loop or pathway) is of paramount importance to identify key elements, develop drugs and design interventions able to compromise network’s functioning, [7].

Among obligate pathogens, *M. tuberculosis* exhibits the highest ratio between number of sigma factors and genome size [8], thus indicating that mechanisms regulating sigma factors availability in *M. tuberculosis* are extremely complex. Among mycobacterial sigma factors, SigE is the only one belonging to the extracytoplasmic function subfamily (i.e., the subfamily of sigma factors that mediate responses when the cell membrane/periplasm, rather than the cytoplasm, is subjected to stress) which is conserved across the *Mycobacterium* genus. Increases in SigE expression level are associated with exposure to heat shock, Sodium Dodecyl Sulfate (SDS, a detergent used to mimic surface stress) and antibiotics (such as isoniazid and vancomycin), [8]. In addition, SigE has been shown to have a major role in determining the amount of bacterial cells surviving prolonged drug treatment [9] and has been proposed as a switch for dormancy [10].

The primal role of SigE in persistence development is hence an established fact; nevertheless, molecular mechanisms and interactions responsible for mycobacterial persistence are only partially understood [10]. In particular, in spite of the intense research efforts coming from both experimental and theoretical/computational fields, it is still unclear which are the positive feedback loops, present within SigE regulatory network, that are essential for persistence. When addressing the problem from a theoretical/computational perspective, persistence is associated with bistability of the mathematical model describing the stress response network. Indeed, coexistence, in a genetically identical population, of two phenotypes, a stress sensitive phenotype with inactive (i.e., poorly expressed) SigE and a persistent phenotype with active (i.e., highly expressed) SigE, corresponds, in mathematical terms, to coexistence of two stable steady states, one with low SigE expression level and the other with high SigE level. In [11], stability analysis of a mathematical model of SigE regulatory network suggested the anti-sigma factor RseA as an important element in emergence of bistability in mycobacterial stress response. The experimental work [12] also hypothesized that regulation of SigE through the sequestering effects of RseA may facilitate persistence. Inspired by the results of [11] and [12], we further investigate the role of the anti-sigma factor RseA by developing a mathematical model of SigE regulatory network which includes three positive feedback loops and explicitly accounts for RseA degradation. Our analysis confirms the importance of RseA for the emergence of bistability and, additionally, elucidates the critical role played by RseA degradation pathway. Interestingly, our findings are in agreement with recent results [13] showing that stress-dependent degradation of RseA can induce modest activation of SigE response network.

**Fig. 1:**
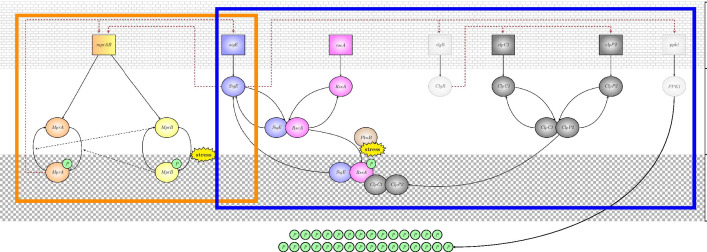
**Functioning of the whole SigE regulatory network.** Red dashed arrows and black solid curved arrows indicate transcriptional and post-translational interactions, respectively. Opaque, light gray color denotes elements (*clgR* mRNA, ClgR protein, *ppk1* mRNA and PPK1 protein) that have not been included in the mathematical model. Orange and blue rectangles highlight the two modules in which the network can be decomposed

### SigE regulatory network

The regulatory network governing SigE availability is schematically reported in Fig. 1. The network is triggered by autophosphorylation of MprB in the presence of polyphosphate (polyP), a linear polymer of orthophosphate whose levels increase following surface and oxidative stress [14], and which serves as a phosphate donor in the MprB autophosphorylation reaction [11]. MprB is a bifunctional enzyme: it can transfer the phosphate to MprA, thereby activating it; but is also capable of dephosphorylating phosphorilated MprA (i.e., MprB acts as a phosphatase in the MprA dephosphorilation reaction) [11]. Both *mprA* and *mprB* genes are cotranscribed from one operon, *mprAB* [11]. Phosphorilated MprA upregulates transcription of the *mprAB* operon. This positive autoregulation of *mprAB* operon due to transcriptional upregulation by phosphorilated MprA gives rise to the first positive feedback loop [11, 15].

The second positive feedback loop arises from transcriptional activation of *sigE* by phosphorylated MprA and subsequent upregulation of *mprAB* transcription from an SigE-dependent promoter [11, 15].

The third positive loop results from the sequence of interactions described in the following. SigE activity is regulated at the post-translational level by the anti-sigma factor RseA which binds to SigE in reducing environments [11, 14]. Thus, RseA reduces SigE availability as transcription factor in the cell since only free SigE upregulates transcription of the *mprAB* operon [11]. RseA, in turn, undergoes phosphorylation-dependant proteolytic degradation in cells subjected to surface stress, but not oxidative stress or heat shock [14]. In particular, RseA degradation pathway is described as follows: in response to surface stress, the serine/threonine protein kinase PknB phosphorylates RseA, which is then targeted by ClpC1P2 for proteolitic degradation, causing the release of the active form of SigE. The increased amount of free SigE results in the induction of the regulon encoding ClgR. Indeed, the expression of ClpC1P2 structural genes is positively controlled by ClgR, whose expression is controlled by SigE [14]. Hence, RseA degradation pathway represents a positive feedback loop: increased amount of free SigE leads to increased amount of ClgR, which positively controls the concentration of ClpC1P2; increased amount of ClpC1P2 in turns results in increased RseA degradation, and hence increased amount of free SigE. In other words, increased amounts of ClpC1P2 leads to more efficient RseA degradation and, consequently, to a higher concentration of free SigE [14].

High polyphosphate intracellular levels are ensured by PPK1, a kinase responsible for polyP biosynthesis and whose transcription is positively regulated by SigE. Hence, increased PPK1 levels raise polyP concentration in the cell, and this in turn stimulates MprA phosphorylation by MprB [6].

### Models of SigE availability in the literature

SigE regulatory network has been the object of experimental and theoretical studies aiming at identification of mechanisms that enable a subset of the bacterial population to persist under stress, see e.g. [9, 12, 16–20]. Sureka and colleagues developed [21] a mathematical model of the stress signalling pathway driven by *mprAB* operon and involving SigE sigma factor, which in turn activates the stringent response regulator Rel. Theoretical analysis predicted bistability in both Rel and SigE expression levels, hinting at phenotypic heterogeneity in a genetically identical cell population. By combining theoretical analysis and single cell analysis by flow cytometry in a *M. smegmatis* population, Sureka and collaborators further showed that a positive feedback loop involving *mprAB* operon along with stochasticity in gene expression are responsible for bimodal distribution of Rel expression levels, and hence for the emergence of bistability.

In a later study [22] by the same research team, unphosphorilated (rather than phosphorylated) MprB is assumed to act as a phosphatase and an additional mechanism leading to bistability is explored. Differently from the model proposed in [21], the revised mathematical model incorporates the effect of growth retardation due to protein synthesis, a mechanism which actually generates a positive feedback. Indeed, a positive feedback loop comes from stress-induced proteins MprA and MprB slowing down cell growth, which results in reduced dilution rate and hence decreased protein decay rate. This reproduces experimental observation that synthesis of stress response protein is accompanied by slower growth rate as compared to non-stress situations. Interestingly, the revised mathematical model exhibits bistability over a larger region of the parameter space with respect to the original model.

Tiwari and colleagues in their work [11] proposed a mathematical model of SigE regulatory network and investigated the mechanisms responsible for the emergence of bistability. In Tiwari’s model the network’s stress response is triggered by autophosphorylation of MprB and subsequent activation of the MprA/MprB two-component system (implementing the first positive feedback loop in SigE regulatory network). MprA/MprB two-component system is a stimulus-response coupling mechanism which controls the ratio between phosphorylated and unphosphorylated portions of MprA and MprB. By analysing the logarithmic gains of the circuit^1^, the Authors showed that the two-component system is not bistable in a biochemically relevant parameter range. Additional regulations are hence included in the model, specifically: transciptional regulation of *sigE* gene by phosphorylated MprA and transciptional regulation of *mprAB* operon by SigE. Again, by analysing the logarithmic gains of the circuit, the Authors were able to show that this extended version of the model exhibits a unique equilibrium point, and hence the second positive feedback loop is insufficient to induce bistability. As a further step, post-translational regulation of SigE by RseA is included in the model. Introduction of this regulation in the network’s model finally leads the system to bistability, which is robust to parameter variation. The Authors hence identified RseA as the key element controlling the ultrasensitive stress response, and predicted that overexpression or deletion of RseA can destroy bistability.

### Paper objective

In this work, we extend Tiwari’s mathematical model and reveal, through theoretical and computational analysis, new insights into bistability of the stress-response network driven by *mprAB* operon and brought about by SigE sigma factor. Our aim is hence twofold: first, to develop a mathematical model of SigE stress response network in *M. tuberculosis* which includes regulations not taken into account by state of the art models (i.e., activation by PknB, regulations mediated by ClpC1P2, and RseA degradation pathway); secondly, to investigate the mechanisms leading to bistability through nullclines analysis and sensitivity analysis.

## Results

**Fig. 2:**
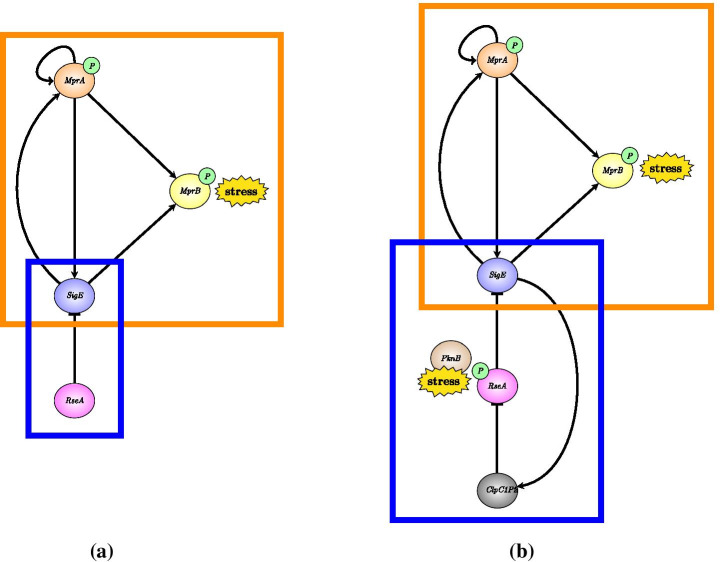
**SigE regulatory network.** (**a**) The model proposed by Tiwari and collaborators provides a dynamic description of the subnetwork highlighted in orange, and considers a constant amount of RseA to capture the effects of the anti-sigma factor. (**b**) Our model extends the model of Tiwari by adding dynamic description of the subnetwork highlighted in blue

Figure 2 illustrates in a simplified and schematic way how the regulatory network of Fig. 1 has been described by Tiwari and colleagues (left) and in the present work (right). The mathematical model proposed by Tiwari and collaborators provides a dynamic description of the subnetwork within the orange rectangle and describes the remaining part of the network (blue rectangle) through the action of a fixed, constant amount of RseA. The mathematical model we are proposing makes a considerable step further by providing a dynamic description also of the subnetwork within the blue rectangle. In particular, our model: (i) describes stress response initiation by both MprB and PknB; (ii) considers regulation in free SigE concentration by ClpC1P2; (iii) accounts for RseA degradation.

The main findings of our work are, firstly, the development of a new model of SigE regulatory network (more accurate with respect to state of the art models) and, secondly, elucidation of the critical role played by RseA degradation pathway for the emergence of bistability and coexistence of two stable equilibria. To disentangle the multiple feedback loops involved within the network, we cast the whole network of Fig. 1 into a block structure with feedback interconnection and adopt an approach based on nullclines analysis to determine, in a reliable though computationally efficient way, the number of equilibrium points of the system.

### Mathematical model

Ordinary differential equations describing the behaviour of MprA/MprB two-component system are borrowed from [11] and reported here for convenience. We adopt capital letters *A* and *B* for state variables representing the concentration level of proteins MprA and MprB, respectively, and we use the subscript *P* to denote the corresponding phosphorylated forms. As in [11], dynamic equations take into account exogenous phosphorylation (dephosphorylation) of MprA (phosphorylated MprA), auto-phosphorylation (auto-dephosphorylation) of MprB (phosphorylated MprB), phosphotransfer from phosphorylated MprB to MprA, phosphatase activity of MprB, up-regulation of MprA and MprB synthesis by phosphorylated MprA and SigE (denoted with the capital letter *E*), and proteins degradation. The resulting differential equations are given by (see Additional file 1 - Supplementary Material for further details):

1 $$\begin{aligned} \frac{dA_P}{dt}&= \frac{k_t}{K_T}AB_P - \frac{k_p}{K_P}A_PB + k_{exp}A - k_{exd}A_P - k_{pdeg}A_P \end{aligned}$$

2 $$\begin{aligned} \frac{dB_P}{dt}&= k_{ap}B - k_{ad}B_P - \frac{k_t}{K_T}AB_P - k_{pdeg}B_P \end{aligned}$$

3 $$\begin{aligned} \frac{dA}{dt}&= \beta _1 \frac{\left( 1 + f_1\frac{A_P^2}{K_1}\right) }{\left( 1 + \frac{A_P^2}{K_1}\right) } + \beta _2 \frac{\left( 1 + f_2\frac{E}{K_2}\right) }{\left( 1 + \frac{E}{K_2}\right) } + \frac{k_p}{K_P}A_PB +\nonumber \\&\qquad - \frac{k_t}{K_T}AB_P + k_{exd}A_P - k_{exp}A - k_{pdeg}A \end{aligned}$$

4 $$\begin{aligned} \frac{dB}{dt}&= \lambda \beta _1 \frac{\left( 1 + f_1\frac{A_P^2}{K_1}\right) }{\left( 1 + \frac{A_P^2}{K_1}\right) } + \lambda \beta _2 \frac{\left( 1 + f_2\frac{E}{K_2}\right) }{\left( 1 + \frac{E}{K_2}\right) } + \frac{k_t}{K_T}AB_P \nonumber \\&\qquad + k_{ad}B_P - k_{ap}B - k_{pdeg}B \end{aligned}$$

The amount of free SigE (denoted by *E*) is described by differential equation (5), which takes into account: positive regulation on the synthesis of SigE by phosphorylated MprA, binding by RseA to form the complex denoted by [*ER*] and dissociation of the [*ER*] complex (here, $R_T$ denotes the total amount of RseA), ClpC1P2-mediated dissociation of the complex $[ER^PC]$ formed by SigE and phosphorylated RseA ($R^P$), protein’s degradation:

5 $$\begin{aligned} \frac{dE}{dt} = \beta _3 \frac{\left( 1 + f_3\frac{A_P^2}{K_1}\right) }{\left( 1 + \frac{A_P^2}{K_1}\right) } - k_3 E R_T + k_4 [ER] + k_8[ER^PC] - k_{pdeg}E \end{aligned}$$

The concentrations of phosphorylated PknB and its unphosphorylated form (denoted by $P^P$ and *P*, respectively) are described by the following differential equations:

6 $$\begin{aligned} \frac{dP^P}{dt}&= k_{ap}^{Pk}P - k_{ad}^{Pk}P^P + k_2 [ERP^P] - k_1 [ER] P^P - k_{pdeg}P^P \end{aligned}$$

7 $$\begin{aligned} \frac{dP}{dt}&= - k_{ap}^{Pk}P + k_{ad}^{Pk}P^P + k_5 [ERP^P] + \nu _P - k_{pdeg}P \end{aligned}$$

The above equations, directly derived from chemical reactions, assume constant PknB synthesis (at rate $\nu _P$) and account for auto-phosphorylation (auto-dephosphorylation) of PknB (phosphorylated PknB), formation and dissociation of the proteins complex degrading RseA (see Additional file 1 - Supplementary Material for further details).

Dynamics of the complex formed by SigE and RseA (denoted by [*ER*]), and of the other complexes involved in RseA degradation pathway (specifically, $[ERP^P]$, $[ER^P]$, $[ER^PC]$) are described by the following differential equations (obtained from the corresponding chemical reactions, see Additional file 1 - Supplementary Material for further details):

8 $$\begin{aligned} \frac{d[ERP^P]}{dt}&= k_1 [ER] P^P - k_2 [ERP^P] - k_5 [ERP^P] - k_{pdeg}[ERP^P] \end{aligned}$$

9 $$\begin{aligned} \frac{d[ER]}{dt}&= k_2 [ERP^P] - k_1 [ER] P^P + k_3 E R_T - k_4 [ER] - k_{pdeg}[ER] \end{aligned}$$

10 $$\begin{aligned} \frac{d[ER^P]}{dt}&= k_5 [ERP^P] - k_6 [ER^P] C + k_7 [ER^PC] - k_{pdeg}[ER^P] \end{aligned}$$

11 $$\begin{aligned} \frac{d[ER^PC]}{dt}&= k_6 [ER^P] C - k_7 [ER^PC] - k_8 [ER^PC] - k_{pdeg} [ER^PC] \end{aligned}$$

Finally, dynamic description of proteins ClpC1, ClpP2 and of the complex ClpC1P2 (denoted by $C_1$, $P_2$ and *C*, respectively) is obtained by taking into account positive regulation on the synthesis of ClpC1 and ClpP2 by SigE, formation and dissociation of the ClpC1P2 complex and of the other complexes (i.e., $[ER^P]$ and $[ER^PC]$) involved in RseA proteolitic degradation. The resulting differential equations are given by:

12 $$\begin{aligned} \frac{dC}{dt}&= - k_6 [ER^P] C + k_7 [ER^PC] + k_8 [ER^PC] + k_9 C_1 P_2 - k_{10}C - k_{pdeg}C \end{aligned}$$

13 $$\begin{aligned} \frac{dC_1}{dt}&= f_{C_1}(E) - k_9 C_1 P_2 + k_{10} C - k_{pdeg}C_1 \end{aligned}$$

14 $$\begin{aligned} \frac{dP_2}{dt}&= f_{P_2}(E) - k_9 C_1 P_2 + k_{10} C - k_{pdeg}P_2 \end{aligned}$$

where $f_{C_1}(E)$ and $f_{P_2}(E)$ denote Hill equations, namely

$$\begin{aligned} f_{C_1}(E)&:= \beta _{C1} \frac{\left( 1 + f_{C1}\frac{E}{K_{C1}}\right) }{\left( 1 + \frac{E}{K_{C1}}\right) }\\ f_{P_2}(E)&:= \beta _{P2} \frac{\left( 1 + f_{P2}\frac{E}{K_{P2}}\right) }{\left( 1 + \frac{E}{K_{P2}}\right) } \end{aligned}$$

Differential equations (1)–(14) describe the functioning of the whole SigE regulatory network under the simplifying assumption, borrowed from Tiwari’s model [11], that RseA concentration is constant. RseA is indeed a fixed parameter of the network, denoted by $R_T$ and appearing in differential equations (5) and (9).

In contrast with the assumption of constant RseA concentration, experimental data show that, in response to surface stress, *rseA* mRNA levels remain stable while RseA concentration decreases [14, 18]. To take into account experimental evidence of non-constant RseA concentration, we further develop an alternative model of SigE regulatory network by considering RseA as a state variable endowed with a proper dynamics. Specifically, we describe RseA concentration by the following differential equation:

15 $$\begin{aligned} \frac{dR}{dt} = \nu _R - k_3 E R + k_4 [ER] - \delta _R R \end{aligned}$$

The above equation accounts for RseA production and degradation (at constant rates $\nu _R$ and $\delta _R$, respectively), formation and dissociation of the complex formed by SigE and RseA. Most importantly, since RseA is proteoliticallty degraded by ClpC1P2, RseA is not retrieved after dissociation of the complex $[ER^PC]$ (differently from what happens to SigE and ClpC1P2, see the terms $+k_8[ER^PC]$ appearing in Eqs. (5) and (12)). The alternative mathematical model of SigE regulatory network, which takes into account RseA dynamics, is hence composed of differential equation (15) together with differential equations (1)–(14), upon substitution of parameter $R_T$ with the state variable *R* in Eqs. (5) and (9).

### Parameters setting

Parameters that define regulatory interactions included in the mathematical model by Tiwari and collaborators have been set in accordance with [11]. Numerical values of these parameters is taken from Table S3 in [11] with two exceptions. First, in our model, to ensure perfect balance between exogenous phosphorylation and dephosphorylation fluxes, the exogenous dephopshorylation rate constant of phosphorylated MprA (i.e., parameter $k_{exd}$) equals the exogenous phopshorylation rate constant of MprA (i.e., parameter $k_{exp}$). This choice is consistent with the analysis carried out in Section 2.5 of [11] and demonstrating bistability of mycobacterial stress-response network (bifurcation diagrams of Figure 5 in [11] have indeed been obtained for $k_{exd} = k_{exp}$). Secondly, for the model with constant RseA concentration, parameter $R_T$ is slightly increased with respect to the value reported in Table S3 of [11] (however, it still belongs to the bistability region reported in Figure S2 of [11]). This modification is justified by the fact that, with respect to the model in [11], we are considering additional proteins complexes in which RseA is present.

Unfortunately, for parameters defining the regulatory interactions mediated by PknB, ClpC1 and ClpP2 (not included in [11]), estimates based on experimental data are not available in the literature. When similar interactions (namely, similar chemical reactions) are described in [11], then corresponding parameters take similar values, see, e.g., the basal transcription rate and the amplification gain of proteins ClpC1, ClpP2. When this reasoning is not applicable, namely, when a parameter playing a similar role in the model by Tiwari cannot be found, then the order of magnitude is set either to $10^{-3}$ (like parameter $\frac{k_p}{K_P}$ in [11]) or to $10^{-2}$ (like parameter $\frac{k_t}{K_T}$ in [11]).

Numerical values of all model parameters, either borrowed from [11] or set according to the previous reasoning, can be found in the Additional file 2 - Table I. In addition, to mitigate the effects due to uncertainties on model parameters for which experimental data are not available, numerical experiments have been performed (see “Parameters perturbation experiments” section) in which parameters whose value is not borrowed from [11] are randomly perturbed, either one at a time or multiple parameters at the same time.

### Bistability investigation through nullclines analysis

**Fig. 3:**
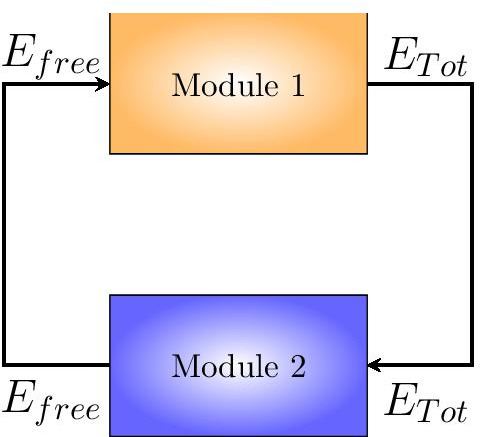
**Feedback interconnection of SigE regulatory network.** (*E* stands for SigE)

The whole SigE regulatory network can be seen as the feedback interconnection of two modules controlling the overall SigE concentration and the amount of total SigE that is free and hence functionally active. This interpretation is graphically illustrated in the block diagram of Fig. 3. In **Module 1** SigE regulation takes place via the two-component system MprA/MprB: free sigma factor SigE (the input to Module 1) regulates transcription of the *mprAB* operon, and hence the total amount of proteins MprA and MprB. The two-component system then controls the ratio between phosphorylated and unphosphorylated portions of MprA and MprB. Only phosphorylated MprA upregulates transcription of *sigE* gene, and thus controls the total amount of SigE protein, which represents the output of Module 1. In **Module 2** the amount of free SigE is controlled by the anti-sigma factor RseA and by proteins ClpC1 and ClpP2. SigE, playing the role of input to Module 2, is partly bound by RseA and degraded by the protein complex ClpC1P2. The remaining free, and hence functionally active, amount of SigE represents the output of Module 2. Clearly, the feedback interconnection is such that the output of Module 1 is the input of Module 2 and, viceversa, the output of Module 2 is the input to Module 1.

In order to underpin the mechanisms leading to coexistence of two stable equilibrium states, we artificially break down the feedback interconnection and derive input–output relationships of each module separately. Via nullclines computation and involved manipulations of their nonlinear algebraic expressions, we provide (i) for Module 1, exact implicit expression of total SigE concentration as a function of free SigE, and (ii) for Module 2, approximated implicit expressions of total SigE concentration as a function of free SigE. We remark that the obtained input–output relationship for Module 2 is not the exact input–output relationship but an approximation of the exact relationship. Indeed, the high non-linearity of the differential equations makes exact computation of the input–output relationship impracticable. However, an accurate approximation of the true input–output relationship can be derived by exploiting time scale separation between proteins degradation and protein complexes formation and dissociation. Then, the number of intersection points between the input–output relationship from Module 1 and the approximated input–output relationship from Module 2 is precisely the number of equilibrium points of the system. Notice that an advantage of our approach based on nullclines analysis is the fact that it allows, for a given set of parameters, to immediately check whether the system admits a unique equilibrium or multiple equilibrium points, without the need to run hundreds of simulations starting from initial conditions that explore, with sufficiently dense sampling, the state space.

**Fig. 4:**
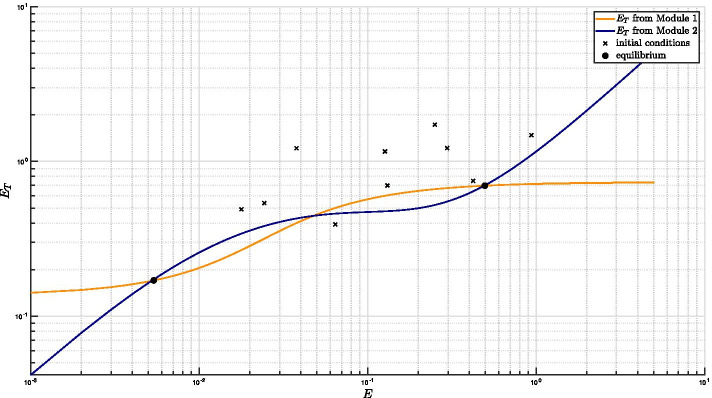
**Nullclines plot under the assumption of non-constant RseA concentration.** When RseA concentration is subject to proteolitic degradation by ClpC1P2, the number of intersection points between the input–output relationships from Module 1 and from Module 2 shows that the closed-loop system is bistable. For the ease of readability, the logarithmic scale has been adopted for *x*- and *y*-axis. *E* stands for SigE

**Fig. 5:**
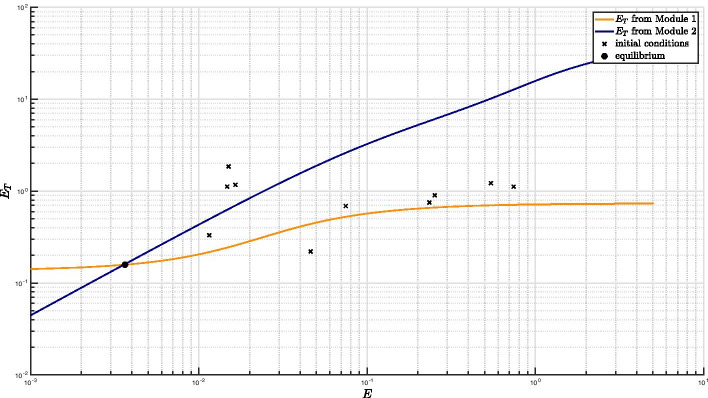
**Nullclines plot under the assumption of constant RseA concentration.** Under the assumption of constant RseA concentration, the number of intersection points between the input–output relationships from Module 1 and from Module 2 implies that the closed-loop system exhibits a unique equilibrium point. For the ease of readability, the logarithmic scale has been adopted for *x*- and *y*-axis. *E* stands for SigE

Clearly, the feedback architecture of Fig. 3 is maintained independently of the regulations included within Module 2. In Tiwari’s model [11] a fixed, constant amount of RseA controls the level of free SigE and represents the unique regulatory mechanism accounted for by Module 2. On the contrary, in our model PknB and ClpC1P2 mediate additional regulations on SigE, and the anti-sigma factor RseA is endowed with a proper dynamics. The choice of relaxing the assumption of constant RseA is justified by the data showing that in response to surface stress, while *rseA* mRNA levels remain stable [24], RseA is proteolitically degraded by ClpC1P2 after its phosphorylation by PknB clearly indicating that in these conditions RseA concentration decreases [14, 18]. On the other hand, from a mathematical point of view, disregarding RseA dynamics and considering it as a fixed parameter means that the positive feedback loop implemented by ClpC1P2 degrading phosphorylated RseA has no actual effect on RseA. For these reasons, we decided to remove the assumption of constant RseA and instead to consider it as a state variable endowed with a proper dynamics. The ordinary differential equation describing RseA concentration accounts for its basal production and degradation (at constant rates), and additional proteolitic degradation by ClpC1P2. Nullclines reported in Fig. 4 clearly show that, under the assumption of dynamic RseA, the closed-loop system exhibits three distinct equilibria, two of which are asymptotically stable (while the third one is necessarily unstable). Remarkably, when the level of RseA is kept constant and the same values (borrowed, when applicable, from [11]) for the remaining model parameters are assumed, the system exhibits a unique equilibrium point^2^, as reported in Fig. 5.

It is worth mentioning that high non-linearity of the mathematical model makes exact analytical derivation of the system’s nullclines impracticable. As a consequence, the curves of Figs. 4 and 5 have been obtained under biologically reasonable approximations (see “Methods” section and Additional file 1 - Supplentary Material for further details on the derivations). Validity and accuracy of the approximated nullclines is however testified by the exact equilibrium points reported with black circles in the figures and obtained by numerical simulations of the model with random initial conditions (indicated with black crosses in the figures). In fact, the equilibrium point obtained via numerical simulation lay at the intersection of the two approximated nullclines.

Figures 4 and 5 together point out the importance of taking into account RseA dynamics and, in particular, its degradation pathway implementing a positive feedback loop on SigE regulation. Looking at the curves^3^ we realize that, in order for the equilibrium point corresponding to higher SigE levels to appear, the blue nullcline needs to undergo a slowdown so as to form, in logarithmic scale, a sort of “plateau”. This is exactly the role of RseA degradation pathway. On the other hand, in order for the equilibrium point corresponding to lower SigE levels to appear, the total amount of SigE (on the vertical axis) needs to be highly sensitive to small variations in free SigE concentrations (on the horizontal axis), so as for the blue nullcline to exhibit a high slope (in logarithmic scale) in its initial segment, i.e., for small amount of free SigE. Our results suggest that a fine regulation of RseA concentration levels is critical for the coexistence of two stable equilibrium points.

### Parameters’ perturbation experiments

Nullclines analysis and model simulations show the need to include RseA degradation pathway into SigE regulatory network’s model in order to capture coexistence of two steady states as experimentally observed. To further investigate the role played by interactions newly introduced into our mathematical model, we perform some numerical experiments in which we perturb model’s parameters and quantitatively evaluate robustness of bistability. When intersection points among nullclines are spaced and clearly distinct, it is reasonable to expect that bistability will be retained in spite of perturbations on the parameters. We hence quantify robustness of bistability by computing the projection on the $E_{free}$-axes of the distance between intersection points corresponding to stable equilibria. Referring to the nullclines plot in Fig. 4 and denoting by $E_1$ and $E_2$ the abscissa of the stable equilibrium points, we introduce the scalar distance function ${\mathcal {D}}(\mathbf{p }) := E_2 - E_1$, where $\mathbf{p }$ is the vector of parameters. Clearly, when monostability arises, such a distance vanishes, i.e., ${\mathcal {D}}(\mathbf{p }) = 0$, since the two stable equilibria coincide and $E_1 = E_2$. We denote by $\bar{{\mathcal {D}}}$ the distance computed with nominal values for the system’s parameters. For an $\varepsilon$-percentage variation on parameters selected through the vector $\mathbf{v }$ whose entries are either $-1$, 0, or 1, we define the following measure of bistability robustness:

16 $$\begin{aligned} {\mathcal {R}}_\varepsilon (\mathbf{v }) := \frac{{\mathcal {D}}(\mathbf{p } + \varepsilon \mathbf{v }) - \bar{{\mathcal {D}}}}{\bar{{\mathcal {D}}}} * \frac{100}{\varepsilon } \end{aligned}$$

Note that efficient computation of the above metric is made possible by the derivation of the (implicit or explicit) solutions to the system’s nullclines.

Since our focus is on the regulations mediated by PknB, ClpC1P2 and RseA degradation pathway, in the following only parameters defining these interactions are perturbed, while model parameters that correspond to analogous parameters in Tiwari’s model [11] and whose value has been borrowed from, are kept constant.

#### Robustness experiment 1

**Fig. 6:**
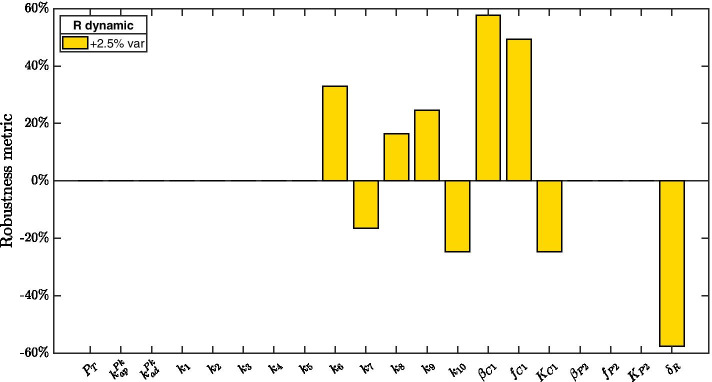
**Robustness experiment 1.** Robustness metric computed after perturbing, one at a time, each parameter by $+2.5\%$

In the first robustness experiment we aim to investigate the effects of local, i.e., with $\varepsilon$ small ($\varepsilon = 2.5\%$) perturbations on the system’s parameters. To this aim, we vary model parameters one at a time, namely we set $\mathbf{v } = \mathbf{e }_i$ where $\mathbf{e }_i$ is the canonical vector with the *i*-th entry equal to 1 and all other entries equal to 0, and we compute the robustness measure ${\mathcal {R}}_{2.5}(\mathbf{e }_i)$. Numerical results obtained by performing such an experiment on the mathematical model with dynamic (i.e., non-constant) RseA concentration are reported in Fig. 6. It results that robustness of bistability is most sensitive to parameters controlling basal and enhanced production of protein ClpC1 (i.e., parameters $\beta _{C1}$ and $f_{C1}$) and to RseA degradation rate (i.e., parameter $\delta _R$). Indeed, a slight increase in ClpC1 production (either basal or enhanced by SigE) substantially increases the robustness metric ${\mathcal R}_{2.5}$. Conversely, an increase in RseA degradation rate causes a decrease in the distance metric $\mathcal D$.

#### Robustness experiment 2

The second robustness experiment is designed to test the effects of random perturbations that (i) have large absolute value (so as to explore regions of the parameter space farther from approximately linear effects explored in Robustness experiment 1), and (ii) involve more that one parameter at a time. For fixed large value of the perturbation $\varepsilon$ the procedure outlined in Algorithm 1 is carried out for the mathematical model including RseA degradation pathway.

*Algorithm 1*

0. Fix the number *N* of perturbations tests and set $i = 1$.
1. Generate a random vector $\mathbf{v }$ whose entries are integers drawn from the discrete uniform distribution on the set $\{-1, 0,1\}$.
2. Compute the distance metric ${\mathcal {D}}(\mathbf{p } + \varepsilon \mathbf{v })$.
3. If $i<N$, repeat from step 1.
4. Plot a histogram of the normalized distance between stable equilibria^4^, i.e., ${\mathcal {D}}(\mathbf{p } + \varepsilon \mathbf{v }) / \bar{{\mathcal {D}}}$.

**Fig. 7:**
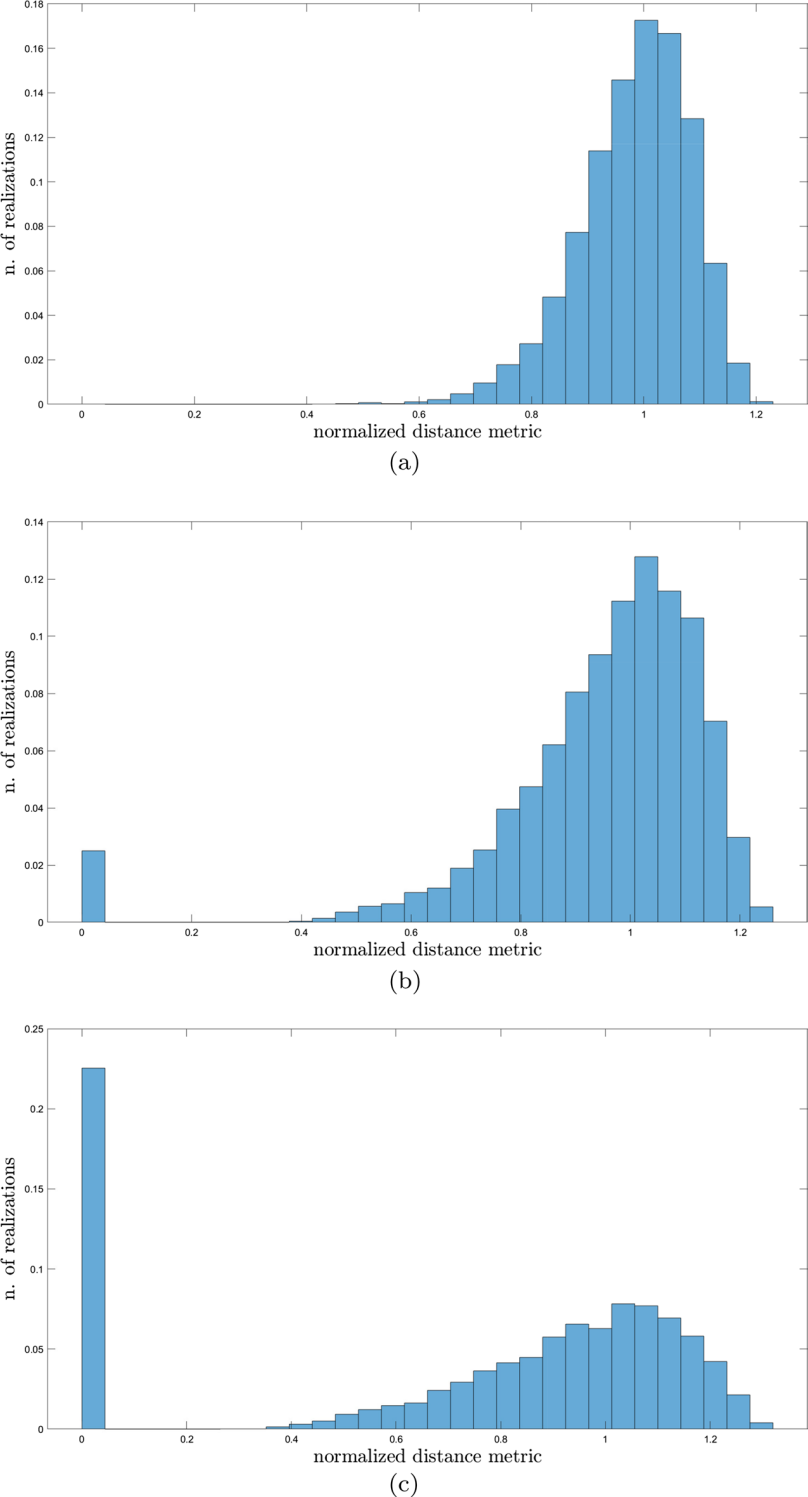
**Robustness experiment 2: normalized distance.** Histogram approximating the relative probability of the normalized distance between equilibria after randomly perturbing parameters as in Algorithm 1 with $N=10^4$ and $\varepsilon = 10\%$ (**a**), $\varepsilon = 15\%$ (**b**), $\varepsilon = 25\%$ (**c**). The height of each bar is $c_i/N$, where $c_i$ is the number of elements in the *i*th bin

The resulting histograms provide an estimate of the relative probability of the distance ${\mathcal {D}}(\mathbf{p } + \varepsilon \mathbf{v })$. Figure 7 illustrates results for $N=10^4$ and percentage variations $\varepsilon = 10\%$ (Fig. 7a), $\varepsilon = 15\%$ (Fig. 7b) and $\varepsilon = 25\%$ (Fig. 7c). It is worth highlighting that bistability is always retained with random perturbations of $\pm 10\%$ with respect to nominal values; when very large perturbations are considered ($\pm 25\%$), bistability is lost on $22.54\%$ of iterations. These results indicate that our mathematical model is quite robust to parameter variations.

**Fig. 8:**
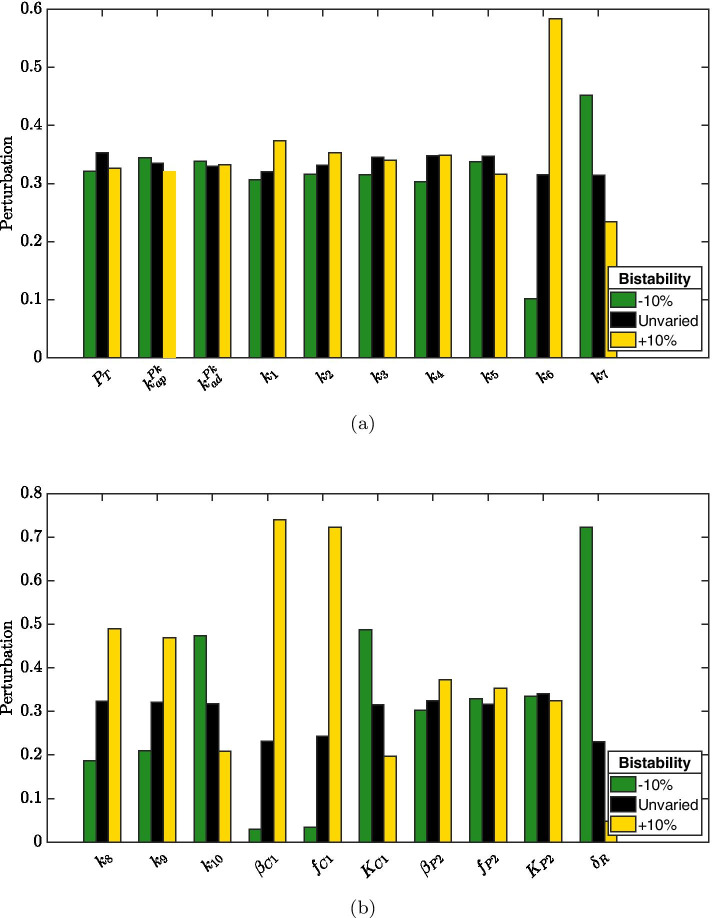
**Robustness experiment 2: parameters variations leading to increased bistability.** For perturbation experiment 2 with $\varepsilon =10\%$, perturbation vectors corresponding to normalised distance greater than 1.1 have been retrieved and their sign patterns collectively reported with bars plot: green bars denote $-10\%$ variation, black bars denote no variation, yellow bars denote $+10\%$ variation

**Fig. 9:**
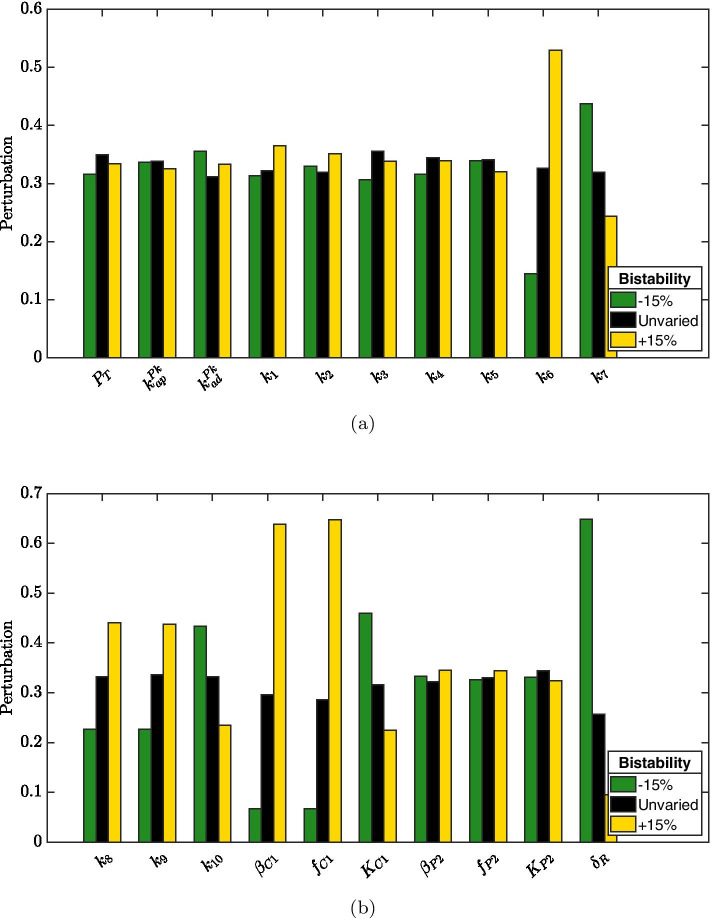
**Robustness experiment 2: parameters variations leading to increased bistability.** For perturbation experiment 2 with $\varepsilon =15\%$, perturbation vectors corresponding to normalised distance greater than 1.1 have been retrieved and their sign patterns collectively reported with bars plot: green bars denote $-15\%$ variation, black bars denote no variation, yellow bars denote $+15\%$ variation

To identify parameters more closely connected to enhanced bistability, we retrieved from perturbation experiments with $\varepsilon = 10\%$ and $\varepsilon = 15\%$ all perturbation vectors $\mathbf{v }$ corresponding to normalized distance metric larger than 1.1. Bar plots summarizing their sign patterns are reported in Figs. 8 and 9. Interestingly, observations resulting from perturbation experiment 1 are confirmed: increased bistability is associated with increased ClpC1 production (either basal or enhanced by SigE) as well as with decreased RseA degradation rate.

**Fig. 10:**
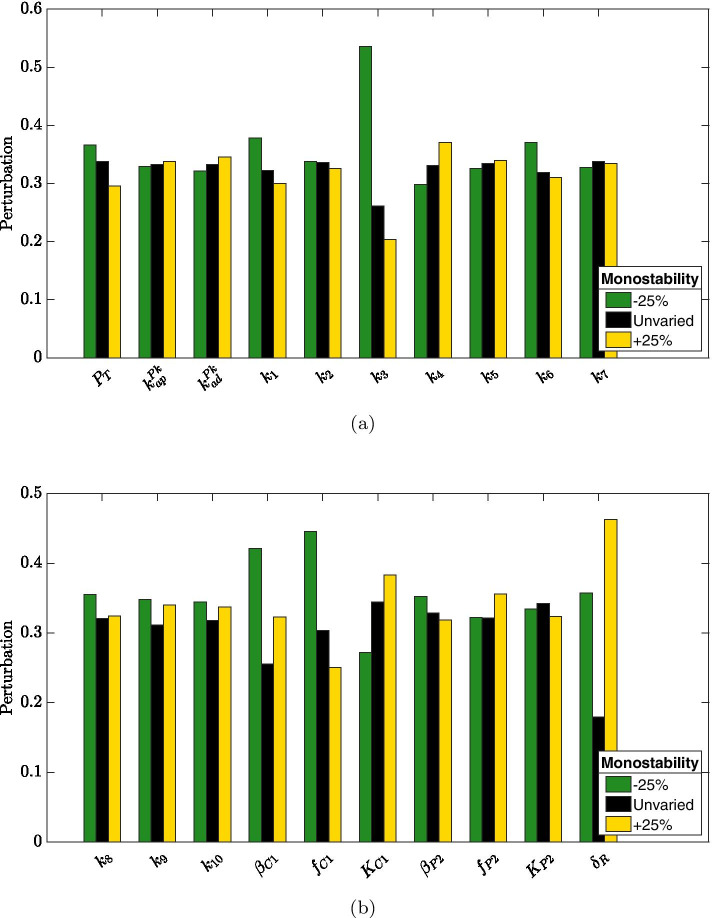
**Robustness experiment 2: parameters variations leading to monostability.** For perturbation experiment 2 with $\varepsilon = 25\%$, perturbation vectors corresponding to a loss of bistability have been retrieved and their sign patterns collectively reported with bars plot: green bars denote $-25\%$ variation, black bars denote no variation, yellow bars denote $+25\%$ variation

To unravel parameters whose variation leads to a loss of bistability, we retrieved from perturbation experiment with $\varepsilon = 25\%$ all perturbation vectors $\mathbf{v }$ corresponding to monostability, whose sign patterns are reported in Fig. 10. Consistently with previous observations, decreases in parameters $\beta _{C1}$ and $f_{C1}$ (which regulate ClpC1 production) and increases in parameter $\delta _R$ are frequently reported when bistability is lost. However, RseA binding rate to SigE (i.e., parameter $k_3$) appears to predominantly control the loss of bistability, its decrease being associated with monostability.

## Discussion

In this work a new mathematical model of SigE regulatory network in *M. tuberculosis* has been proposed and fruitfully exploited to investigate the mechanisms responsible for bistability. Our model considerably extends the model proposed by Tiwari and collaborators in [11] in the sense that, starting from the same mathematical description of the chemical reactions (i.e., the same set of ODEs), additional transcriptional ad post-translational regulations have been included. These regulations implement an extra positive feedback loop, and hence play a potential crucial role in controlling network’s response to stress. In particular, differently from what done in [11], we consider two distinct mechanisms triggering the network’s response to stress: exogenous phosphorylation of MprB and PknB-dependant phosphorylation of RseA. This latter event is responsible for the release of free SigE resulting from RseA proteolitic degradation by ClpC1P2, whose expression is, in turn, (indirectly) positivelely controlled by free SigE. In addition, since, as shown in [11], RseA is a key element in controlling the stress response, we further investigate its role by removing the simplifying assumption (introduced in [11]) of constant concentration and by taking into account proteolitic degradation by ClpC1P2.

To efficiently investigate under what conditions two stable equilibria coexist, we make use of model’s nullclines whose expressions have been derived, through involved computations on the ODEs, either explicitly or, when this was not possible, implicitly. The proposed approach boasts two advantageous features over the solution via numerical integration of the differential equations: first, given a set of parameters, a single run of the script program (rather than thousands of simulations) allows to assess whether the system is bistable or monostable; secondly, sampling of initial conditions over the state space is averted, thus bypassing the risk of missing an equilibrium point due to incomplete sampling of the parameter space.

Plotting the nullclines for both the mathematical model with constant RseA concentration and the model incorporating RseA degradation pathway, some interesting remarks can be made. First of all, the two models with common parameters assuming identical values exhibit qualitatively different behaviours. Indeed, taking into account RseA dynamic evolution leads the system to switch from monostability to bistability. Interestingly, our findings are in agreement with recent results [13] reporting that (modest) activation of MprA-SigE network is achieved through stress-dependent degradation of RseA. Specifically, Rao and collaborators construct a *M. tuberculosis* strain with suppressed MprB autokinase activity (through overexpression of the chaperone protein Dnak), and show that, following exposure to SDS, *mprA* gene is modestly upregulated while *sigE* mRNA is not significantly increased. Rao and colleagues hence hypothesize that degradation of RseA may be responsible, through release of SigE, for the modest activation of the MprA-SigE network. Modeling results [13] support this hypothesis and additionally show that induction of *mprA* is lost when stress-dependent degradation of RseA is not present in the model. The results of our analysis go in the same direction by showing that RseA degradation pathway is crucial for the activation of the stress response and hence for the emergence of bistability.

Robustness of the bistability property is investigated by introducing random perturbations on the parameters. Specifically, a perturbation experiment is performed in which parameters defining regulations mediated by PknB, ClpC1P2 and RseA degradation are slightly varied (i.e., by $2.5\%$ of the nominal value) one at a time. In addition, to further explore bistability robustness, we consider random perturbations larger in absolute value ($\pm 10\%$, $\pm 15\%$ and $\pm 25\%$ of the nominal value) and affecting multiple parameters simultaneously. It results that bistability property is retained in spite of random perturbations up to $\pm 10\%$ of the nominal values. This means that bistability is a robust feature of our model. On the other hand, it is easy to imagine how bistability can be beneficial for a bacterial population as it allows some cells to enter a persistence state more easily or quickly than others. In other words, a fraction of cells can face exposure to stress by expressing the SigE regulon at maximum levels, while the remaining fraction can express SigE at a reduced level. This heterogeneity allows cells to specialize in advance if circumstances should mutate again: the cells of the second group might have lower chances to survive if the level of stress is very high, but those that survive might be able to quickly resume growth after cessation of stress; conversely, cells of the first group are likely to survive high levels of stress, but might require more time to recover. Each of these two populations is thus specialized to increase the efficiency according to changing environmental conditions.

**Fig. 11:**
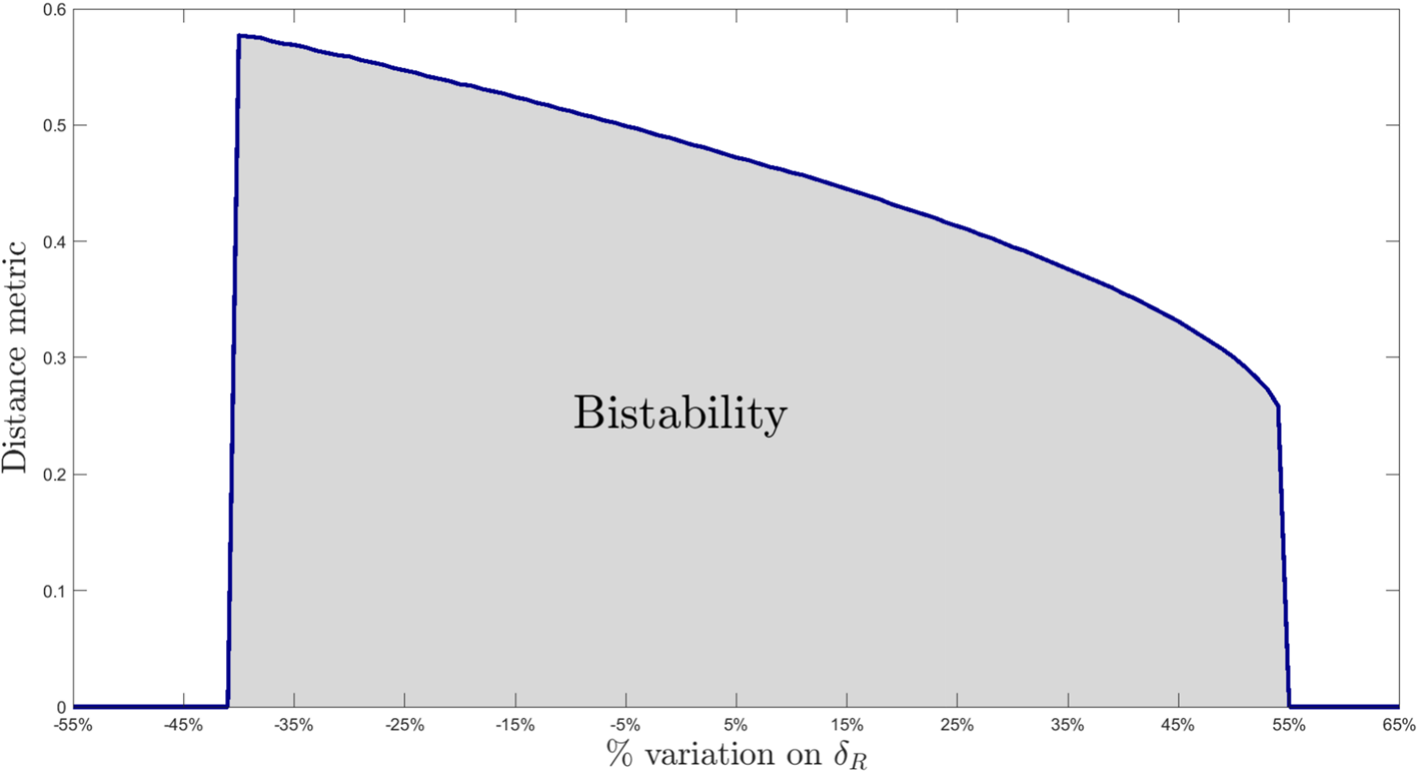
**Bistability dependence on RseA degradation rate.** Distance between stable equilibria computed after varying RseA degradation rate only

Perturbation experiments also allow to identify parameters (and hence, from the chemical reactions they represent, the molecular interactions) that crucially control the emergence of bistability. The production of protein ClpC1 seems to predominantly influence the distance between the two stable equilibria: increasing ClpC1 production, either basal ($\beta _{C1}$) or enhanced by SigE ($f_{C1}$), results in increased bistability. Not surprisingly, RseA degradation rate ($\delta _R$) remarkably affects bistability: a slight increase in RseA degradation rate (i.e., by $2.5\%$) leads to decreased distance between stable equilibria, and large increases (i.e., by $25\%$) are frequently reported when bistability is lost. Recalling that the system exhibits a unique equilibrium point when RseA concentration is kept constant, namely when RseA degradation rate is arbitrarily small, the fact that increased RseA degradation is associated to reduced bistability points to a fine control of RseA expression levels. To further explore this aspect we singularly perturbed RseA degradation rate (letting all other parameters assume their nominal value) and we computed the distance between stable equilibria. The obtained curve reported in Fig. 11 shows that both extremal conditions result in monostability, while only intermediate values of RseA degradation rate lead to bistaility. The proposed analysis suggests that precise regulation of RseA concentration could be a hallmark of a bistable SigE regulatory network and a determinant factor for the *M. tuberculosis* to adapt to stress situations.

It is worth mentioning that the mathematical model we have developed, whilst representing a considerable extension of the model proposed in [11], still relies on some simplifying assumptions that limit its applicability. First, for the sake of nullclines’ analytical tractability, we made the implicit assumption that free SigE directly upregulates transcription of proteins ClpC1 and ClpP2. Actually, this regulation is mediated by ClgR, an intermediate step that necessarily introduces a temporal delay on ClpC1 and ClpP2 production as well as on ClpC1P2 complex formation. Since time delays are, generally speaking, associated with bistability, we expect the qualitative behaviour of the model to remain unchanged and our considerations to hold true in spite of the simplifying assumption on ClpC1P2 regulation. Secondly, our model does not include positive regulation mediated by ClgR on PPK1, which is responsible for polyphosphate biosynthesis. This simplification is justified by the realistic assumption that polyphosphate are most abundant, and hence always available for proteins phosphorylation.

## Conclusions

State of the art models [11] focus on stress response initiation by MprB and on SigE regulation by MprA/MprB two-component system (transcription level) and by RseA (post-translation level). Other mechanisms exist that trigger stress response and control the amount of free SigE, and these interactions may be important for the emergence of bistability. Differently from state of the art models, the model we are proposing takes into account additional interactions, it is hence more realistic from a biological point of view and able to provide a more accurate description of the network. In particular, key features of our mathematical model are: (i) description of stress response initiation by both MprB and PknB; (ii) regulation in free SigE concentration by ClpC1P2 (implementing a positive feedback loop); (iii) account for RseA degradation. These characteristics allow us to investigate at a deeper level the mechanisms responsible for the emergence of bistability. In particular, we have shown that taking into account RseA degradation pathway is crucial in order for the model to exhibit two distinct stable equilibria. Perturbation experiments on the model’s parameters pointed out the predominant role played by production of protein ClpC1 both at basal rate and at enhanced (by SigE) rate. In addition, our results collectively prove that in order for the network to exhibit bistability, RseA concentration needs to be finely controlled.

## Methods

We first extend Tiwari’s model of SigE stress response network by including PknB- and ClpC1P2-mediated regulations and by removing the assumption of constant RseA concentration. Secondly, we investigate the mechanisms leading to bistability through nullclines analysis and manipulations, and we perform perturbation experiments in order to identify parameters that crucially control the emergence of bistable behaviours.

Ordinary Differential Equations (ODEs) describing the network's dynamics have been obtained starting from chemical reactions and under the assumption of quasi-steady state approximation for mRNA dynamics. This assumption is justified by the fact that mRNA dynamics is faster than proteins’ dynamics (see Additional file 1  - Supplementary Material for further details). The developed state-space model is then regarded as the composition of two modules in feedback interconnection with total SigE concentration and amount of free SigE playing the role of input and output. Via nullclines computation and involved manipulations of their nonlinear algebraic expressions, we artificially break down the feedback interconnection and derive input–output relationships of the two modules separately. The analysis is carried out first under the assumption of constant RseA concentration and then in the more realistic setting in which RseA is subject to ClpC1P2-mediated degradation.

### Nullclines analysis

In order to determine the number of equilibrium points of the ODE system (1)–(14), we propose an approach based on nullclines analysis. Nullclines are, indeed, an effective tool to assess the number of equilibria of a system: the number of equilibrium points is precisely given by the number of points where all of the nullclines intersect. A clear advantage of this technique is that it does not require running multiple simulations with initial conditions sampled over the state space of the system. Nullclines analysis is hence less computationally demanding and, what is more, not affected by sampling of the initial conditions over the state-space. On the contrary, when investigating system’s stability through model simulations, two distinct equilibria might exist but sufficiently dense sampling over the right area of the state space is required in order for the equilibrium points to be detected.

Unfortunately, when dealing with nonlinear systems of high (or relatively high) dimension, as the model (1)–(14), determining the number of solutions of the algebraic equations system is not obvious at all. To address the problem we recast the whole SigE network model (1)–(14) into the feedback architecture of Fig. 3, artificially break down the interconnection between Module 1 and Module 2, and derive input–output relationships of the two open-loop systems separately. Specifically, for each module, we first compute nullclines and manipulate them so as to obtain a suitable system of algebraic equations. Secondly, considering free SigE (i.e., state variable *E* of the mathematical model) as the independent variable, we rewrite the algebraic system in such a way that every nullcline is (directly or indirectly) a function, either exact or approximated, of *E*. This allows to compute (i) for Module 1, the total amount of SigE as an exact, implicit function of free SigE; and (ii) for Module 2, the total amount of SigE as an approximated function of free SigE. For Module 2, the analysis is carried out first under the assumption of constant RseA concentration (parameter $R_T$) and then in the more realistic setting in which RseA is endowed with a proper dynamics (state variable *R*).

Due to space limitations and in order to keep the analysis more easily understandable, we report here only the final (implicit or explicit) solution of the system’s nullclines. All detailed computations and manipulations leading to the their derivations, together with the approximations introduced for Module 2, can be found in Additional file 1 - Supplementary Material.

#### Module 1: nullclines solution in terms of *E*

The amount of free SigE represents the input to Module 1, which controls the total SigE concentration (representing the output of Module 1) through MprA/MprB two-component system. The static input–output relationship for Module 1 is:

$$\begin{aligned} E_T = \frac{{\mathcal H}_3(\phi ^{-1}(E))}{k_{pdeg}} \end{aligned}$$

where ${\mathcal H}_3(\phi ^{-1}(E)) = {\mathcal H}_3(A_P)$ is the Hill function describing positive regulation by phosphorylated MprA on *mprAB* operon, i.e.,

$$\begin{aligned} {\mathcal H}_3(A_P) = \beta _3 \frac{\left( 1 + f_3\frac{A_P^2}{K_1}\right) }{\left( 1 + \frac{A_P^2}{K_1}\right) } \end{aligned}$$

and $A_P = \phi ^{-1}(E)$ is the inverse of the function

$$\begin{aligned} \phi (A_P) := K_2 \frac{\frac{k_{pdeg}}{\beta _2} \left\{ A_T^+(A_P) - \frac{{\mathcal H}_1(A_P)}{k_{pdeg}} \right\} - 1}{f_2 - \frac{k_{pdeg}}{\beta _2} \left\{ A_T^+(A_P) - \frac{{\mathcal H}_1(A_P)}{k_{pdeg}} \right\} } \end{aligned}$$

**Fig. 12:**
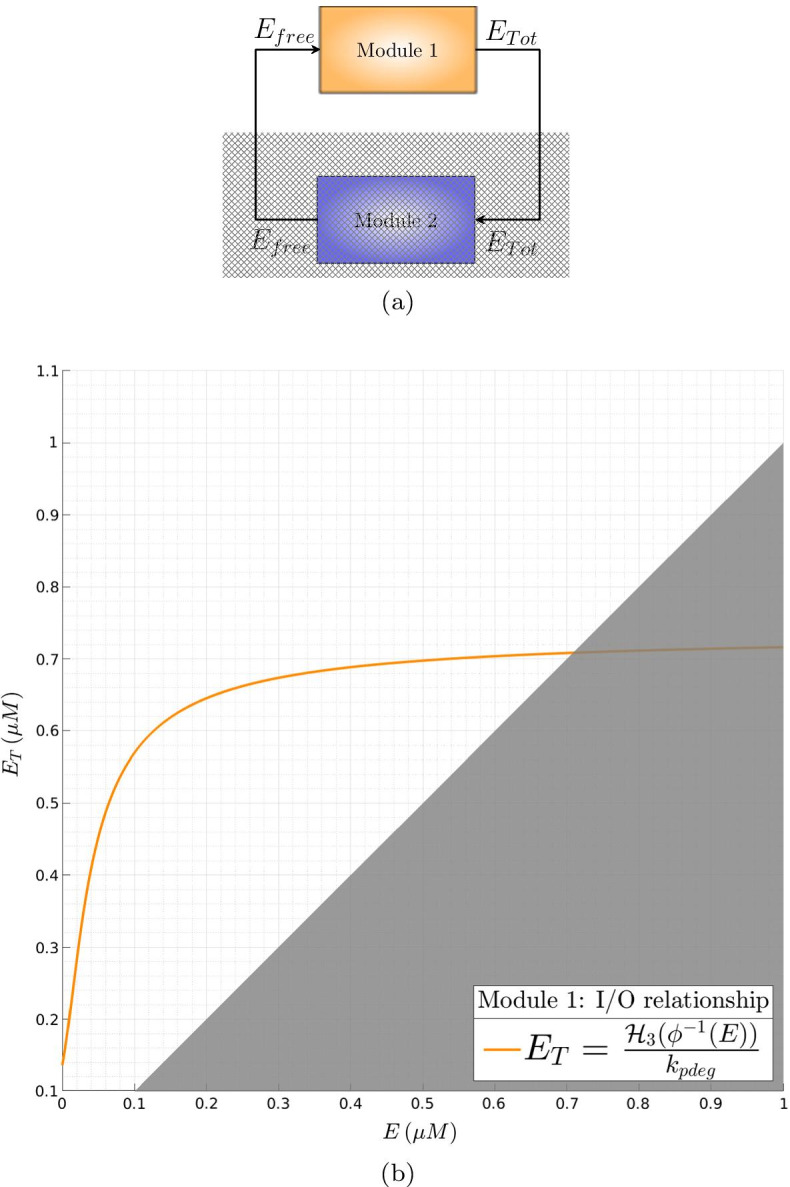
**Input–output relationship for Module 1.** (**a**) Block diagram. (**b**) Nullcline associated to the subsystem composed of ODEs (1)–(4) together with the ODE obtained by summing up Eqs. (5) and (8)–(11). On the *x*- and *y*-axis are reported the amount of free SigE and the total concentration of SigE, respectively. The shading removes a region without biological meaning, where free SigE would be larger that total SigE .

Unfortunately, it is not possible to derive en explicit, closed form expression of the function $\phi ^{-1}(E)$, and hence only an implicit expression can be provided for the static input–output relationship of Module 1. Since the ordinary differential equations describing the functioning of Module 1 are borrowed from [11], the curve we obtainded, plotted in Fig. 12, is consistent with the blue curve named “autoregulation module” reported in Figure 4 of [11].

#### Module 2: nullclines solution in terms of *E* under the assumption of constant $R_T$

Module 2 captures regulations on SigE, whose total concentration represents the module’s input, that control the amount of free, and hence functionally active, SigE. Since multiple proteins and complexes are involved in these regulations, the analysis of Module 2 is considerably more involved than that of Module 1, and it only allows for the derivation of approximated explicit expression of total SigE concentration as a function of free SigE. Specifically, approximated nullclines expressions are obtained by exploiting the fact that protein degradation rate (parameter $k_{pdeg}$) is significantly smaller than kinetic parameters regulating protein complexes formation and dissociation (e.g., parameters $k_4$ and $k_{10}$). Note that also Tiwari and collaborators [11], when deriving the ordinary differential equation system, exploited time scale separation between slow protein degradation and fast post-translational interactions (see [11], Section 1.2 of the Supplementary Material).

Without going deeply into details (which can be found in Additional file 1 - Supplementary Material), we report here the final equations expressing, for each complex including SigE, the steady-state concentration as a function of *E*.

The complex obtained from RseA binding to SigE reaches the steady-state value:

17 $$\begin{aligned}{}[ER] = \frac{ -{\mathcal B}(E) + \sqrt{{\mathcal B}^2(E) - 4 \cdot A \cdot {\mathcal C}(E)} }{2 \cdot A} \end{aligned}$$

where ${\mathcal B}(E)$ and ${\mathcal C}(E)$ are functions of *E* given respectively by

$$\begin{aligned} {\mathcal B}(E)&:= \frac{1}{R_T} \frac{k_5}{k_3} k_1 k_{ap}^{Pk} P_T + \frac{1}{R_T} \frac{k_4}{k_3} \left( k_{ap}^{Pk} + k_{ad}^{Pk}\right) (k_2 + k_5) - k_1 \left( k_5 + k_{ap}^{Pk}\right) E \\ {\mathcal C}(E)&:= - \left( k_{ap}^{Pk} + k_{ad}^{Pk}\right) (k_2 + k_5) E \end{aligned}$$

By exploiting equation (17), the steady-state concentration of the complex formed when phosphorylated PknB binds to [*ER*] can be expressed as:

18 $$\begin{aligned}{}[ERP^P] = \frac{k_1 k_{ap}^{Pk} P_T [ER]}{ \left( k_{ap}^{Pk} + k_{ad}^{Pk}\right) (k_2 + k_5) + k_1 \left( k_5 + k_{ap}^{Pk}\right) [ER]} \end{aligned}$$

where $P_T$ is the total amount of PknB, $P_T = P^P + P + [ERP^P]$.

Expression (18) allows to compute the nullcline associated with the protein complex ClpC1P2:

$$\begin{aligned} C = \frac{k_{10}}{k_9} \frac{1}{4} \left( -1 + \sqrt{1 + 4 \frac{k_9}{k_{10}} h(E)} \right) ^2 - \frac{k_{pdeg}}{k_{10}} \frac{k_5}{k_8} [ERP^P] \end{aligned}$$

where the function *h*(*E*) is given by

$$\begin{aligned} h(E) = \frac{f_{C1}(E)}{k_{pdeg}} - \frac{k_5}{k_8} [ERP^P] \end{aligned}$$

The steady-state value of the complex formed from SigE and phosphorylated RseA is given by

19 $$\begin{aligned}{}[ER^P] = \frac{ \frac{k_5}{k_8} [ERP^P]}{ \frac{k_6}{k_7 + k_8} C + \frac{k_{pdeg}}{k_8}} \end{aligned}$$

The nullcline associated to the complex formed when ClpC1P2 binds to $[ER^P]$ is given by

20 $$\begin{aligned}{}[ER^PC] = \frac{C}{C + k_{pdeg} \frac{k_7 + k_8}{k_8 k_6}} \frac{k_5}{k_8} [ERP^P] \end{aligned}$$

**Fig. 13:**
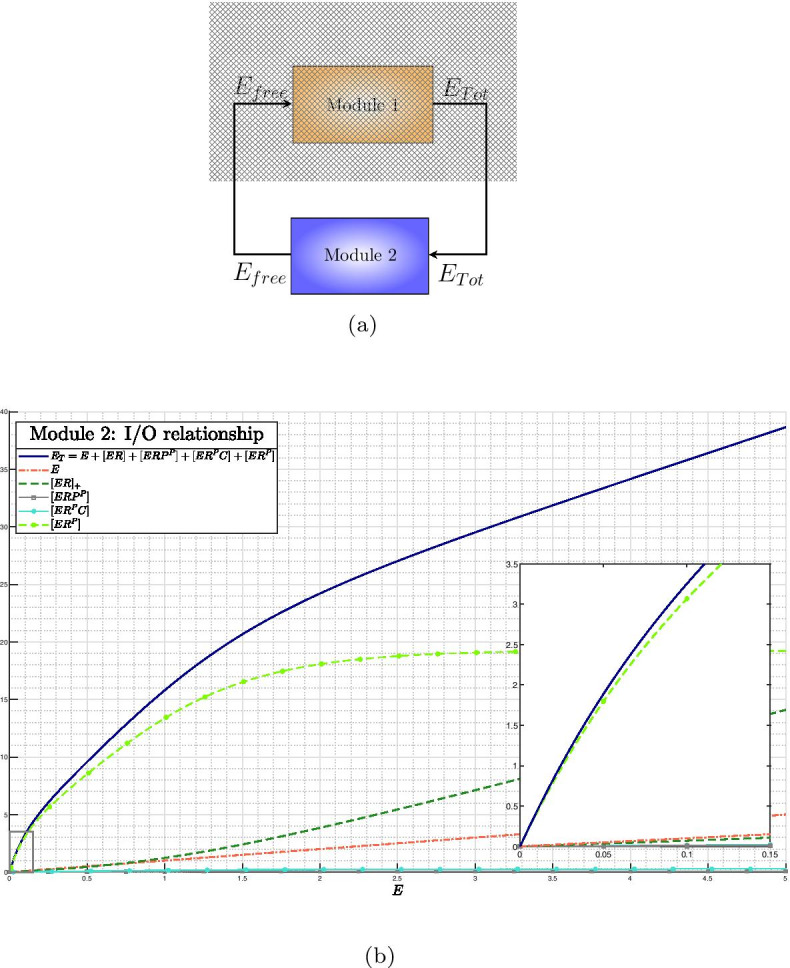
**Input–output relationship for Module 2.** (**a**) Block diagram. (**b**) Nullclines associated to the subsystem composed of ODEs (5)–(14). The blue thick line represents the amount of total SigE as a function of free SigE (reported on the *x*-axis). Total SigE concentration has been computed as the summation of free SigE (dashed red line) and complexes in which SigE is bounded by RseA and/or ClpC1P2 (various colours and traits). Inset: magnification of the area within the gray rectangle

Finally, since the total amount of SigE is given by $E_T := E + [ER] + [ER^P] + [ER^PC] + [ERP^P]$, by putting together Eqs. (17)–(20), the input–output relationship for Module 2 can be obtained. In Fig. 13 all components contributing to the total amount of SigE, i.e., *E*, [*ER*], $[ER^P]$, $[ER^PC]$, $[ERP^P]$, are plotted as functions of *E*, together with the input–output relationship for Module 2.

By putting together the input–output relationship from Module 1 (reported in Fig. 12) and that from Module 2 (reported in Fig. 13), it can be concluded that, under the assumption of constant RseA concentration, the closed-loop system exhibits a unique equilibrium point, as previously shown in Fig. 5.

#### Module 2: nullclines solution in terms of *E* when *R* is a dynamic variable

Upon defining $R_{max}= \frac{\nu _R}{\delta _R}$, the following explicit solution for [*ER*] nullcline can be obtained:

21 $$\begin{aligned}{}[ER] = \frac{ -{\mathcal B}(E) + \sqrt{{\mathcal B}^2(E) - 4 \cdot A \cdot {\mathcal C}(E)} }{2 \cdot A} \end{aligned}$$

where we have set $\alpha _n := k_1 k_{ap}^{Pk} P_T$, $\alpha _d := k_1 \left( k_5 + k_{ap}^{Pk}\right)$, $\beta _d := \left( k_{ap}^{Pk} + k_{ad}^{Pk} \right) \left( k_2 + k_5\right)$, $A := \frac{1}{R_{max}} \frac{k_4}{k_3} \alpha _d$, and the functions ${\mathcal B}(E)$ and ${\mathcal C}(E)$ are given respectively by

$$\begin{aligned} {\mathcal B}(E) :&= \frac{1}{R_{max}} \frac{k_5}{k_3} \alpha _n + \frac{1}{R_{max}} \frac{k_4}{k_3} \beta _d - \left( \alpha _d - \frac{1}{\delta _R R_{max}} k_5 \alpha _n \right) E\\ {\mathcal C}(E) :&= - \beta _d E \end{aligned}$$

The above formulation highlights the role played by $R_{max}$ and $\delta _R$: when $R_{max} = R_T$ and $\delta _R \rightarrow +\infty$, the above quadratic equation in [*ER*] reduces to the analogous quadratic equation (17) obtained under the assumption of constant RseA concentration ($R \equiv R_T$). On the contrary, when $R_{max} < R_T$ and $\delta _R < +\infty$, both the constant coefficient *A* and the function ${\mathcal B}(E)$ are larger than the corresponding terms previously derived.

Nullclines associated with complexes $[ER^P]$, $[ER^PC]$ and $[ERP^P]$ can then be computed from Eqs. (19)–(20) by keeping in mind the new expression for [*ER*] provided in Eq. (21).

**Fig. 14:**
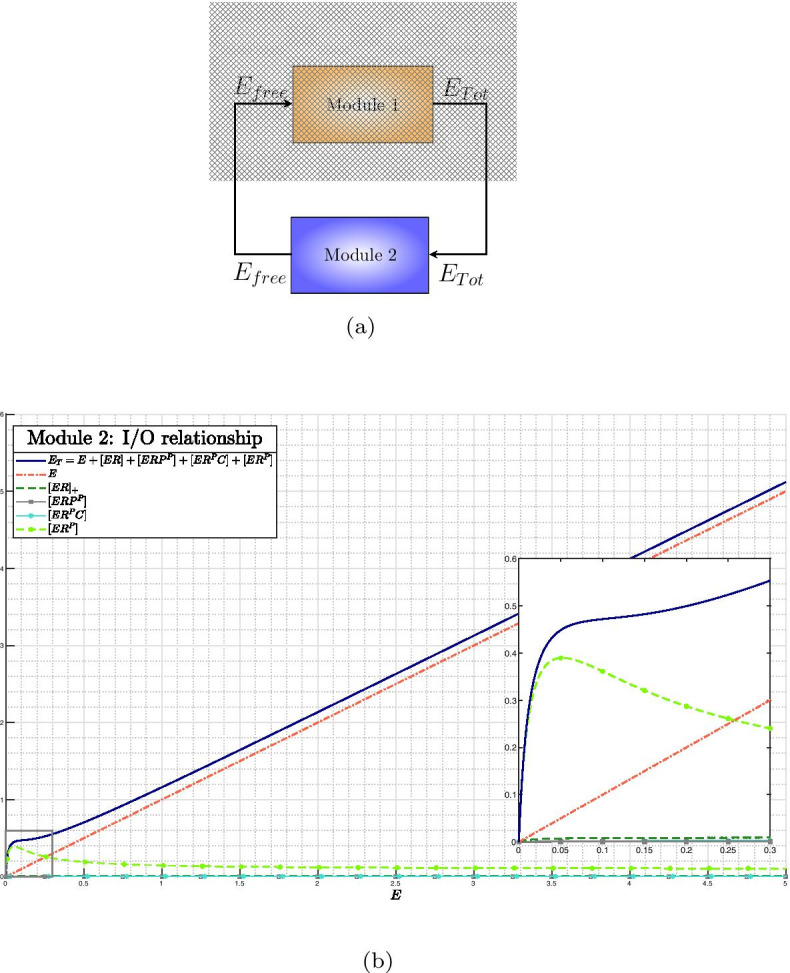
**Input–output relationship for Module 2 under the assumption of non-constant RseA concentration.** (**a**) Block diagram. (**b**) Nullclines of the subsystem obtained from differential Eq. (15) together with ODEs (5)–(14) upon substitution of $R_T$ with *R*. The blue thick line represents the amount of total SigE as a function of free SigE (reported on the *x*-axis). Total SigE concentration has been computed as the summation of free SigE (dashed red line) and complexes in which SigE is bounded by RseA and/or ClpC1P2 (various colours and traits). Inset: magnification of the area within the gray rectangle

Nullclines associated with complexes contributing to the overall amount of SigE are reported in Fig. 14, together with the resulting input–output relationship for Module 2.

Putting together the input–output relationships from Module 1 (reported in Fig. 12) and the input–output relationship from Module 2 (reported in Fig. 14), it can be concluded (see Fig. 4) that, under the more realistic working conditions of dynamic RseA concentration, the closed-loop system exhibits two clearly distinct equilibrium points.

## Supplementary information

**Additional file 1 - Supplementary Material**. This document lists chemical reactions described by the mathematical model, contains information on model development and additional model simulations, provides detailed nullclines derivation.

**Additional file 2 - Table I**. This table contains numerical values of model parameters.

## Data Availability

Data sharing is not applicable as no datasets were generated nor analysed in the study. MATLAB code used to generate presented results is available from the corresponding author on reasonable request.

## Footnotes

1 As explained in [11], logarithmic gains are the effective kinetic orders (i.e., the exponents) of the power-law system representation proposed by Savageau in [23].

2 Both this last simplified model and the model in [11] share the assumption of constant RseA, but SigE–RseA interaction is described in a very different way. Therefore it is not surprising that the first predicts bistability while the latter predicts a unique equilibrium point.

3 Note that the plots of Figs. 4 and 5 do not report temporal dynamics but static relationships between total amount of SigE and amount of free SigE.

4 For the ease of interpretation, histogram plots are also normalized to the total number of iterations, namely we plot $c_i/N$, where $c_i$ is the number of elements in the *i*th bin.
