# Supplementary material for: Mathematical modelling of SigE regulatory network reveals new insights into bistability of mycobacterial stress response
Source: BMC Bioinformatics. 2021 Nov 19;22:558. doi: 10.1186/s12859-021-04372-5 (PMC8605609; doi:10.1186/s12859-021-04372-5)
Supplement: Supplementary file 1 — Additional file 1 - Supplementary Material. This document lists chemical reactions described by the mathematical model, contains information on model development and additional model simulations, provides detailed nullclines derivation. [file 12859_2021_4372_MOESM1_ESM.pdf]

Supplementary Material for "Mathematical modelling of SigE regulatory network reveals new insights into bistability of mycobacterial stress response"

Irene Zorzan, Simone Del Favero, Alberto Giaretta, Riccardo Manganelli, Barbara Di Camillo, Luca Schenato

1. Chemical reactions

The following chemical reactions represent the starting point for the derivation of a set of Ordinary Differential Equations describing stress response of SigE regulatory network. Chemical reactions not included in the model proposed by Tiwari and collaborators in [1] are marked with a diamond.

- Exogenous phosphorylation and dephosphorylation of MprA:
  $A \underset{k_{exd}}{\overset{k_{exp}}{\rightleftharpoons}} A^P$
- Autophosphorylation and autodephosphorylation of MprB:
  $B \underset{k_{ad}}{\overset{k_{ap}}{\rightleftharpoons}} B^P$
- Phosphotransfer reaction from phosphorylated MprB to MprA:
  $A + B^P \underset{k_{d1}}{\overset{k_{b1}}{\rightleftharpoons}} A|B^P \xrightarrow{k_t} A^P + B$
- MprB-mediated dephosphorylation of phosphorylated MprA:
  $A^P + B \underset{k_{d2}}{\overset{k_{b2}}{\rightleftharpoons}} A^P|B \xrightarrow{k_p} A + B$
- SigE production:
  $\emptyset \xrightarrow{f_E(A^P)} E$
- MprA production:
  $\emptyset \xrightarrow{f_A(A^P,E)} A$
- MprB production:
  $\emptyset \xrightarrow{\lambda f(A^P,E)} B$
- RseA binding to SigE: complex formation and dissociation
  $E + R \underset{k_4}{\overset{k_3}{\rightleftharpoons}} E|R$

◇ Autophosphorylation and autodephosphorylation of PknB:
  $PknB \underset{k_{ad}^{Pk}}{\overset{k_{ap}^{Pk}}{\rightleftharpoons}} PknB^P$

◇ PknB-mediated phosphorylation of RseA:
  $E|R + PknB^P \underset{k_2}{\overset{k_1}{\rightleftharpoons}} E|R|PknB^P \xrightarrow{k_5} E|R^P + PknB$

◇ RseA proteolitic degradation by ClpC1P2:
  $E|R^P + C1|P2 \underset{k_7}{\overset{k_6}{\rightleftharpoons}} E|R^P|C1|P2 \xrightarrow{k_8} E + R + C1|P2$

◇ ClpC1 production:
$\emptyset \xrightarrow{f_{C_1}(ClgR,E)} C1$

◇ ClpP2 production:
$\emptyset \xrightarrow{f_{P_2}(ClgR,E)} P2$

◇ ClpC1P2 complex formation and dissociation:
$C1 + P2 \underset{k_{10}}{\overset{k_9}{\rightleftharpoons}} C1|P2$

2. Mathematical model

Under the assumptions of quasi-steady state approximation for mRNA dynamics, the functioning of SigE regulatory network is described by the following set of ODEs:

$$\frac{dA_P}{dt} = \frac{k_t}{K_T}AB_P - \frac{k_p}{K_P}A_PB + k_{exp}A - k_{exd}A_P - k_{pdeg}A_P \quad (1)$$

$$\frac{dB_P}{dt} = k_{ap}B - k_{ad}B_P - \frac{k_t}{K_T}AB_P - k_{pdeg}B_P \quad (2)$$

$$\frac{dA}{dt} = \beta_1\frac{\left(1+f_1\frac{A_P^2}{K_1}\right)}{\left(1+\frac{A_P^2}{K_1}\right)} + \beta_2\frac{\left(1+f_2\frac{E}{K_2}\right)}{\left(1+\frac{E}{K_2}\right)} + \frac{k_p}{K_P}A_PB - \frac{k_t}{K_T}AB_P + k_{exd}A_P - k_{exp}A - k_{pdeg}A \quad (3)$$

$$\frac{dB}{dt} = \lambda\beta_1\frac{\left(1+f_1\frac{A_P^2}{K_1}\right)}{\left(1+\frac{A_P^2}{K_1}\right)} + \lambda\beta_2\frac{\left(1+f_2\frac{E}{K_2}\right)}{\left(1+\frac{E}{K_2}\right)} + k_{ad}B_P - k_{ap}B + \frac{k_t}{K_T}AB_P - k_{pdeg}B \quad (4)$$

$$\frac{dE}{dt} = \beta_3\frac{\left(1+f_3\frac{A_P^2}{K_1}\right)}{\left(1+\frac{A_P^2}{K_1}\right)} - k_3ER_T + k_4[ER] + k_8[ER^PC] - k_{pdeg}E \quad (5)$$

$$\frac{dP^P}{dt} = k_{ap}^{Pk}P - k_{ad}^{Pk}P^P + k_2[ERP^P] - k_1[ER]P^P - k_{pdeg}P^P \quad (6)$$

$$\frac{dP}{dt} = -k_{ap}^{Pk}P + k_{ad}^{Pk}P^P + k_5[ERP^P] + \nu_P - k_{pdeg}P \quad (7)$$

$$\frac{d[ERP^P]}{dt} = k_1[ER]P^P - k_2[ERP^P] - k_5[ERP^P] - k_{pdeg}[ERP^P] \quad (8)$$

$$\frac{d[ER]}{dt} = k_2[ERP^P] - k_1[ER]P^P + k_3ER_T - k_4[ER] - k_{pdeg}[ER] \quad (9)$$

$$\frac{d[ER^P]}{dt} = k_5[ERP^P] - k_6[ER^P]C + k_7[ER^PC] - k_{pdeg}[ER^P] \quad (10)$$

$$\frac{d[ER^PC]}{dt} = k_6[ER^P]C - k_7[ER^PC] - k_8[ER^PC] - k_{pdeg}[ER^PC] \quad (11)$$

$$\frac{dC}{dt} = -k_6[ER^P]C + k_7[ER^PC] + k_8[ER^PC] + k_9C_1P_2 - k_{10}C - k_{pdeg}C \quad (12)$$

$$\frac{dC_1}{dt} = f_{C_1}(E) - k_9C_1P_2 + k_{10}C - k_{pdeg}C_1 \quad (13)$$

$$\frac{dP_2}{dt} = f_{P_2}(E) - k_9C_1P_2 + k_{10}C - k_{pdeg}P_2 \quad (14)$$

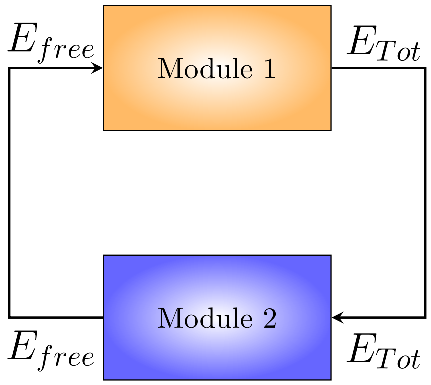

Figure 1: Feedback interconnection of SigE regulatory network.

where

$$f_{C_1}(E) := \beta_{C1} \frac{\left(1 + f_{C1} \frac{E}{K_{C1}}\right)}{\left(1 + \frac{E}{K_{C1}}\right)}$$

$$f_{P_2}(E) := \beta_{P2} \frac{\left(1 + f_{P2} \frac{E}{K_{P2}}\right)}{\left(1 + \frac{E}{K_{P2}}\right)}$$

and v_P is the production rate of P.

3. Bistability investigation through nullclines analysis

Nullclines analysis is a powerful tool to determine the number of equilibrium points of a system: the number of equilibrium points is given by the number of points where all of the nullclines intersect. A clear advantage of this technique is that it does not require running multiple simulations with initial conditions sampled over the state space of the system. On the other hand, when dealing with nonlinear systems of high (or relatively high) dimension, determining the number of solutions of the algebraic equations system is not obvious at all. To overcome this problem we decompose SigE regulatory network into two interconnected modules (see Figure 1):

- In **Module 1** SigE regulation takes place via the two-component system MprA/MprB: free sigma factor SigE (the input to Module 1) regulates transcription of the *mprAB* operon, and hence the total amount of proteins MprA and MprB. The two-component system then controls the ratio between phosphorylated and unphosphorylated portions of MprA and MprB: only phosphorylated MprA upregulates transcription of *sigE*, and thus controls the total amount of SigE, which represents the output of Module 1.
- In **Module 2** the amount of free SigE is controlled by the anti-sigma factor RseA and by proteins ClpC1 and ClpP2: total amount of SigE (playing the role of input to Module 2) is partly sequestered or degraded by these proteins (state variables $[ER]$, $[ER^P]$, $[ERP^P]$ and $[ER^PC]$); the remaining, free SigE represents the output of Module 2.

Note that the output of Module 1 is the input of Module 2 and, viceversa, the output of Module 2 is the input to Module 1. In the following, we artificially break down the interconnection among the two modules, and separately analyze them to obtain input-output relationships. In particular, for each module, we first compute nullclines and manipulate them so as to obtain a suitable system of algebraic equations (Section 3.1 for the MprA/MprB regulation module, Section 3.3 for RseA/ClpC1P2 regulation module). Secondly, considering E as the independent variable, we rewrite the algebraic system in such a way that every nullcline is (directly or indirectly) a function of E (Section 3.2

for the MprA/MprB regulation module, Section 3.4 for RseA/ClpC1P2 regulation module). This allows to compute, for both modules, the total amount of SigE as a function of free SigE. Then, for a given a set of parameters, it is immediate to check whether the system admits a unique equilibrium or multiple equilibrium points.

3.1. Module 1: nullclines computation and derivation of the algebraic equations system

3.1.1. Two-component system MprA/MprB.

Define the total amount of MprA protein as $A_T := A_P + A$, and notice that $\frac{dA_T}{dt} = \frac{dA_P}{dt} + \frac{dA}{dt} = \mathcal{H}_1(A_P) + \mathcal{H}_2(E) - k_{pdeg}A_T$, where we have defined

$$\mathcal{H}_1(A_P) := \beta_1 \frac{1 + f_1 \frac{A_P^2}{K_1}}{1 + \frac{A_P^2}{K_1}}, \qquad \text{and} \qquad \mathcal{H}_2(E) := \beta_2 \frac{1 + f_2 \frac{E}{K_2}}{1 + \frac{E}{K_2}}$$

The nullcline for A_T is hence given by:

$$\boxed{A_T = \frac{1}{k_{pdeg}} \left\{ \mathcal{H}_1(A_P) + \mathcal{H}_2(E) \right\}} \tag{15}$$

Similarly, define $B_T := B_P + B$ and notice that $\frac{dB_T}{dt} = \lambda \mathcal{H}_1(A_P) + \lambda \mathcal{H}_2(E) - k_{pdeg}B_T$, which leads to the nullcline

$$B_T = \frac{\lambda}{k_{pdeg}} \left\{ \mathcal{H}_1(A_P) + \mathcal{H}_2(E) \right\},$$

namely

$$\boxed{B_T = \lambda A_T} \tag{16}$$

Recalling that $A = A_T - A_P$ and $B = B_T - B_P$, the equilibrium conditions on A_P and B_P yields:

$$\frac{k_t}{K_T}(A_T - A_P)B_P - \frac{k_p}{K_P}A_P(B_T - B_P) + k_{exp}(A_T - A_P) - k_{exd}A_P - k_{pdeg}A_P = 0 \tag{17}$$

$$k_{ap}(B_T - B_P) - k_{ad}B_P - \frac{k_t}{K_T}(A_T - A_P)B_P - k_{pdeg}B_P = 0 \tag{18}$$

3.1.2. Total amount of SigE.

Define the total amount of SigE as $E_T := E + [ER] + [ER^P] + [ER^PC] + [ERP^P]$, and notice that:

$$\begin{aligned} \frac{dE_T}{dt} &= \frac{dE}{dt} + \frac{d[ER]}{dt} + \frac{d[ER^P]}{dt} + \frac{d[ER^PC]}{dt} + \frac{d[ERP^P]}{dt} \\ &= \beta_3 \frac{\left(1 + f_3 \frac{A_P^2}{K_1}\right)}{\left(1 + \frac{A_P^2}{K_1}\right)} - k_{pdeg}E_T \end{aligned}$$

The equilibrium condition $\frac{dE_T}{dt} = 0$ leads to the equation

$$\boxed{E_T = \frac{\mathcal{H}_3(A_P)}{k_{pdeg}},} \tag{19}$$

where we have set

$$\mathcal{H}_3(A_P) = \beta_3 \frac{\left(1 + f_3 \frac{A_P^2}{K_1}\right)}{\left(1 + \frac{A_P^2}{K_1}\right)}$$

3.2. Module 1: nullclines solution in terms of E

Plugging equation (16) into equation (18) the following equation is obtained:

$$k_{ap}\lambda A_T - k_{ap}B_P - k_{ad}B_P - \frac{k_t}{K_T}A_T B_P + \frac{k_t}{K_T}A_P B_P - k_{pdeg}B_P = 0$$

Solving the above equation for B_P yields:

$$B_P = \frac{\lambda \overbrace{k_{ap}A_T}^{n(A_T)}}{\underbrace{\frac{k_t}{K_T}A_T - \frac{k_t}{K_T}A_P + k_{ap} + k_{ad} + k_{pdeg}}_{d(A_T,A_P)}} = \lambda \frac{n(A_T)}{d(A_T,A_P)} \tag{20}$$

Now, let us express $B_T - B_P$ as

$$\begin{aligned} B_T - B_P &= \lambda A_T - B_P \\ &= \lambda A_T - \lambda \frac{n(A_T)}{d(A_T,A_P)} \\ &= \lambda \left(\frac{A_T d(A_T,A_P) - n(A_T)}{d(A_T,A_P)} \right) \end{aligned} \tag{21}$$

Plugging equations (20) and (21) into the algebraic equation (17), after tedious computations we obtain a quadratic equation in the unknown A_T of the form:

$$\mathcal{A}(A_P)A_T^2 + \mathcal{B}(A_P)A_T + \mathcal{C}(A_P) = 0,$$

where:

$$\begin{aligned} \mathcal{A}(A_P) &= \lambda \frac{k_t}{K_T}k_{ap} - \lambda \frac{k_p}{K_P}\frac{k_t}{K_T}A_P + k_{exp}\frac{k_t}{K_T} \\ \mathcal{B}(A_P) &= -\lambda \frac{k_t}{K_T}k_{ap}A_P + \lambda \frac{k_p}{K_P}\frac{k_t}{K_T}A_P^2 - \lambda \frac{k_p}{K_P}\left(k_{ad} + k_{pdeg}\right)A_P + \\ &\quad - \frac{k_t}{K_T}k_{exp}A_P + \left(k_{ap} + k_{ad} + k_{pdeg}\right)k_{exp} - \frac{k_t}{K_T}\left(k_{exp} + k_{exd} + k_{pdeg}\right)A_P \\ \mathcal{C}(A_P) &= -\left(k_{exp} + k_{exd} + k_{pdeg}\right)\left(-\frac{k_t}{K_T}A_P + k_{ap} + k_{ad} + k_{pdeg}\right)A_P \end{aligned}$$

Unfortunately, it is not easy to determine the sign of $\mathcal{A}(A_P)$, $\mathcal{B}(A_P)$ and $\mathcal{C}(A_P)$ and hence to decide which of the two solutions is the admissible one. However, plotting A_T as a function of A_P it is immediate to realize that the only solution satisfying the feasibility constraint $A_T \geq A_P$ is:

$$A_T^+(A_P) = \frac{-\mathcal{B}(A_P) + \sqrt{\mathcal{A}^2(A_P) - 4\cdot\mathcal{A}(A_P)\cdot\mathcal{C}(A_P)}}{2\cdot\mathcal{A}(A_P)} \tag{22}$$

Plugging equation (22) into the algebraic equation (15) and solving for E we obtain:

$$E = K_2 \frac{\frac{k_{pdeg}}{\beta_2}\left\{A_T^+(A_P) - \frac{\mathcal{H}_1(A_P)}{k_{pdeg}}\right\} - 1}{f_2 - \frac{k_{pdeg}}{\beta_2}\left\{A_T^+(A_P) - \frac{\mathcal{H}_1(A_P)}{k_{pdeg}}\right\}} =: \phi(A_P),$$

and hence $A_P = \phi^{-1}(E)$ (unfortunately, it is not possible to derive en explicit, closed form expression for $\phi^{-1}(E)$). Finally, from equation (19) it follows that

$$E_T = \frac{\mathcal{H}_3(\phi^{-1}(E))}{k_{pdeg}}$$

which represents the static input-output relationship of Module 1.

3.3. Module 2: nullclines computation and derivation of the algebraic equations system

3.3.1. Subnetwork involving PknB.

Define the total amount of PknB as $P_T := P^P + P + [ERP^P]$, and notice that $\frac{dP_T}{dt} = \frac{dP^P}{dt} + \frac{dP}{dt} + \frac{d[ERP^P]}{dt} = v_P - k_{pdeg}P_T$, hence at equilibrium it holds

$$P_T = \frac{v_P}{k_{pdeg}}$$

The equilibrium condition $\frac{d[ERP^P]}{dt} = 0$ leads to the algebraic equation:

$$\begin{aligned} k_1[ER]P^P &= (k_2 + k_5 + k_{pdeg})[ERP^P] \\ &\simeq (k_2 + k_5)[ERP^P] \end{aligned}$$

where we exploited the fact that $k_{pdeg} \ll k_2, k_5$.

Similarly, the equilibrium condition $\frac{dP^P}{dt} + \frac{d[ERP^P]}{dt} = 0$ yields:

$$(k_5 + k_{pdeg})[ERP^P] = k_{ap}^{Pk}P - (k_{ad}^{Pk} + k_{pdeg})P^P$$

Again, since $k_{pdeg} \ll k_5, k_{ad}^{Pk}$, we will replace the above equation with the following equilibrium condition:

$$k_5[ERP^P] = k_{ap}^{Pk}P - k_{ad}^{Pk}P^P$$

By substituting $P = P_T - P^P - [ERP^P]$, and recalling that $k_1[ER]P^P = (k_2 + k_5)[ERP^P]$, the equilibrium condition results

$$\boxed{[ERP^P]\left\{\left(k_{ap}^{Pk} + k_{ad}^{Pk}\right)(k_2 + k_5) + k_1\left(k_5 + k_{ap}^{Pk}\right)[ER]\right\} = k_1 k_{ap}^{Pk}[ER]P_T} \tag{23}$$

3.3.2. Subnetwork involving the complexes formed by SigE.

The equilibrium condition for $\frac{d[ERP^P]}{dt} + \frac{d[ER]}{dt} = 0$ leads to:

$$\begin{aligned} 0 &= -(k_5 + k_{pdeg})[ERP^P] + k_3 ER_T - (k_4 + k_{pdeg})[ER] \\ &\simeq -k_5[ERP^P] + k_3 ER_T - k_4[ER] \end{aligned}$$

where we exploited the fact that $k_{pdeg} \ll k_4, k_5$.

The equilibrium condition for $\frac{d[ER^P]}{dt} + \frac{d[ER^PC]}{dt} = 0$ yields:

$$\begin{aligned} 0 &= k_5[ERP^P] - (k_8 + k_{pdeg})[ER^PC] - k_{pdeg}[ER^P] \\ &\simeq k_5[ERP^P] - k_8[ER^PC] - k_{pdeg}[ER^P] \end{aligned}$$

Hence, the nullclines result

$$k_5[ERP^P] + k_4[ER] = k_3 ER_T \tag{24}$$

$$[ER^PC] = \frac{k_5}{k_8}[ERP^P] - \frac{k_{pdeg}}{k_8}[ER^P] \tag{25}$$

3.3.3. Subnetwork involving ClpC1.

For the sake of analytical tractability we introduce the simplifying assumption that proteins ClpC1 and ClpP2 are described by identical differential equations (i.e., identical parameters) and exhibit the same initial concentration, so that $C_1(t) = P_2(t)$ for every $t \geq 0$. Define the total amount of ClpC1 protein as $C_T := C_1 + C + [ER^PC]$, and note that:

$$\begin{aligned} \frac{dC_T}{dt} &= \frac{dC_1}{dt} + \frac{dC}{dt} + \frac{d[ER^PC]}{dt} \\ &= f_{C1}(E) - k_{pdeg}C_T \end{aligned}$$

yielding the equilibrium condition

$$C_T = \frac{f_{C1}(E)}{k_{pdeg}}$$

Recalling that $C_1 = P_2$, the equilibrium condition $\frac{d[ER^P C]}{dt} + \frac{dC}{dt} = 0$ leads to:

$$\begin{aligned} 0 &= k_9 C_1^2 - k_{10} C - k_{pdeg}(C + [ER^P C]) \\ &= k_9 C_1^2 - \left(k_{10} + k_{pdeg}\right) C - k_{pdeg}[ER^P C] \\ &= k_9 C_1^2 - k_{10} C - k_{pdeg}[ER^P C] \end{aligned} \tag{26}$$

where we exploited the fact that $k_{pdeg} \ll k_{10}$. Recalling that $C_1 = P_2$, the equilibrium condition for C is given by:

$$\begin{aligned} 0 &= -k_6[ER^P]C + k_7[ER^P C] + k_8[ER^P C] + k_9 C_1^2 - k_{10} C - k_{pdeg} C \\ &= -k_6[ER^P]C + k_7[ER^P C] + k_8[ER^P C] + k_{pdeg}[ER^P C] \\ &= -k_6[ER^P]C + \left(k_7 + k_8 + k_{pdeg}\right)[ER^P C] \\ &\simeq -k_6[ER^P]C + (k_7 + k_8)[ER^P C] \end{aligned}$$

where we first made use of equilibrium condition (26), and then exploited the fact that $k_{pdeg} \ll k_7, k_8$.

So, nullclines associated to the subnetwork involving ClpC1 are given by

$$\boxed{\begin{aligned} C_T &= \frac{f_{C1}(E)}{k_{pdeg}} && (27) \\ k_9 C_1^2 &= k_{10} C + k_{pdeg}[ER^P C] && (28) \\ [ER^P C] &= \frac{k_6}{k_7 + k_8}[ER^P][C] && (29) \end{aligned}}$$

To sum up, nullclines computation yields the following algebraic equations system (for convenience we gather here algebraic equations (23)-(29)):

$$[ERP^P]\left\{\left(k_{ap}^{Pk} + k_{ad}^{Pk}\right)(k_2 + k_5) + k_1\left(k_5 + k_{ap}^{Pk}\right)[ER]\right\} = k_1 k_{ap}^{Pk}[ER]P_T \tag{30}$$

$$k_5[ERP^P] + k_4[ER] = k_3 ER_T \tag{31}$$

$$[ER^P C] = \frac{k_5}{k_8}[ERP^P] - \frac{k_{pdeg}}{k_8}[ER^P] \tag{32}$$

$$C_T = \frac{f_{C1}(E)}{k_{pdeg}} \tag{33}$$

$$C = \frac{k_9}{k_{10}} C_1^2 - \frac{k_{pdeg}}{k_{10}}[ER^P C] \tag{34}$$

$$[ER^P C] = \frac{k_6}{k_7 + k_8}[ER^P][C] \tag{35}$$

3.4. Module 2: nullclines solution in terms of E

The algebraic equations system (30)-(35) is composed of 6 algebraic equations in 7 unknowns (i.e., E, $[ER]$, $[ER^P]$, $[ER^P C]$, $[ERP^P]$, C_1 and C), and hence it is underdetermined. Taking E as the independent variable, we now solve the algebraic system for the remaining unknowns. In other words, we express all of the nullclines (except for E-nullcline) as function of E.

3.4.1. $[ER]$-nullcline as a function of E.

From equations (30)-(31), the following quadratic equation in the unknown $[ER]$ can be obtained:

$$\underbrace{\frac{1}{R_T}\frac{k_4}{k_3}k_1\left(k_5+k_{ap}^{Pk}\right)}_{A}[ER]^2+$$
$$+\underbrace{\left\{\frac{1}{R_T}\frac{k_5}{k_3}k_1k_{ap}^{Pk}P_T+\frac{1}{R_T}\frac{k_4}{k_3}\left(k_{ap}^{Pk}+k_{ad}^{Pk}\right)(k_2+k_5)-k_1\left(k_5+k_{ap}^{Pk}\right)E\right\}}_{\mathcal{B}(E)}[ER]+$$
$$\underbrace{-\left(k_{ap}^{Pk}+k_{ad}^{Pk}\right)(k_2+k_5)E}_{\mathcal{C}(E)}=0$$

Note that, by Descartes' rule of signs, only one of the two solutions is positive, and hence the explicit solution for $[ER]$ is given by:

$$[ER]=\frac{-\mathcal{B}(E)+\sqrt{\mathcal{B}^2(E)-4\cdot A\cdot\mathcal{C}(E)}}{2\cdot A} \tag{36}$$

3.4.2. $[ERP^P]$-nullcline as a function of E.

We now express $[ERP^P]$-nullcline as a function of $[ER]$, which is in turn a function of E by equation (36): from equation (30) it immediately follows[1]

$$[ERP^P]=\frac{k_1k_{ap}^{Pk}P_T[ER]}{\left(k_{ap}^{Pk}+k_{ad}^{Pk}\right)(k_2+k_5)+k_1\left(k_5+k_{ap}^{Pk}\right)[ER]} \tag{37}$$

3.4.3. C, C_1 and $[ER^PC]$-nullclines as a function of E.

Unfortunately, explicit, exact expressions for C, C_1 and $[ER^PC]$-nullclines as a function of E cannot be readily obtained. An iterative procedure converging to the exact solution in few iterations can be designed as follows. First of all, note that from equation (35) $[ER^P]$ can be expressed as

$$[ER^P]=\frac{k_7+k_8}{k_6}\frac{[ER^PC]}{[C]} \tag{38}$$

Plugging (42) into equation (32) and solving for $[ER^PC]$ we obtain:

$$[ER^PC]=\frac{[C]}{[C]+k_{pdeg}\frac{k_7+k_8}{k_8k_6}}\frac{k_5}{k_8}[ERP^P] \tag{39}$$

Substituting (39) into equation (34) yields:

$$C=\frac{k_9}{k_{10}}C_1^2-\frac{k_{pdeg}}{k_{10}}\frac{[C]}{[C]+k_{pdeg}\frac{k_7+k_8}{k_8k_6}}\frac{k_5}{k_8}[ERP^P] \tag{40}$$

Secondly, plugging (33) and (34) into the definition of C_T, we obtain:

$$\begin{aligned}\frac{f_{C1}(E)}{k_{pdeg}}&=C_1+\frac{k_9}{k_{10}}C_1^2-\frac{k_{pdeg}}{k_{10}}[ER^PC]+[ER^PC]\\&=C_1+\frac{k_9}{k_{10}}C_1^2+\left(1-\frac{k_{pdeg}}{k_{10}}\right)[ER^PC]\\&\simeq C_1+\frac{k_9}{k_{10}}C_1^2+[ER^PC]\end{aligned}$$

[1] Alternatively, we can proceed analogously to what done for $[ER]$-nullcline: by combining equations (30) and (31), a quadratic equation in the unknown $[ERP^P]$ can be obtained. However, in this case Descartes' rule of signs implies that both solutions are positive, and it is not easy to decide which of the two solutions is the correct one. We hence prefer expression (37).

where we used the fact that $k_{pdeg} \ll k_{10}$. Upon substituting $[ER^P C]$ with expression (39), the previous equation can be rewritten as

$$\frac{k_9}{k_{10}} C_1^2 + C_1 - \underbrace{\left\{ \frac{f_{C1}(E)}{k_{pdeg}} - \frac{[C]}{[C] + k_{pdeg} \frac{k_7+k_8}{k_8 k_6}} \frac{k_5}{k_8} [ERP^P] \right\}}_{h(E,C)} = 0,$$

Since $h(E,C) := C_T - [ER^P C]$ is non-negative, Descartes' rule of signs guarantees that only one solution of the previous quadratic equation is positive. We hence obtain the following expression for C_1-nullcline as a function of $h(E,C)$:

$$C_1 = \frac{k_{10}}{k_9} \frac{1}{2} \left(-1 + \sqrt{1 + 4 \frac{k_9}{k_{10}} h(E,C)} \right) \tag{41}$$

We now compute C, C_1 and $[ER^P C]$ nullclines with the following iterative procedure:

A0: Set $k = 0$, and initialize $[ER^P C]^{(k)} = \lim_{C \to +\infty} [ER^P C]$ from (39), namely $[ER^P C]^{(0)} = \frac{k_5}{k_8} [ERP^P]$. Then, $h^{(0)}(E,C) = \lim_{C \to +\infty} h(E,C)$, namely:

$$\begin{aligned} h^{(0)}(E,C) &= \frac{f_{C1}(E)}{k_{pdeg}} - [ER^P C]^{(0)} \\ &= \frac{f_{C1}(E)}{k_{pdeg}} - \frac{k_5}{k_8} [ERP^P] \end{aligned}$$

Initialize $C_1^{(k)}$ resorting to equation (41), namely:

$$C_1^{(0)} = \frac{k_{10}}{k_9} \frac{1}{2} \left(-1 + \sqrt{1 + 4 \frac{k_9}{k_{10}} h^{(0)}(E,C)} \right)$$

Initialize $C^{(k)}$ resorting to equation (40) and $C \to +\infty$, namely:

$$\begin{aligned} C^{(0)} &= \frac{k_9}{k_{10}} \left(C_1^{(0)} \right)^2 - \frac{k_{pdeg}}{k_{10}} [ER^P C]^{(0)} \\ &= \frac{k_9}{k_{10}} \left(C_1^{(0)} \right)^2 - \frac{k_{pdeg}}{k_{10}} \frac{k_5}{k_8} [ERP^P] \end{aligned}$$

A1: Set $k = k+1$ and update $[ER^P C]^{(k)}$, $h^{(k)}$, $C_1^{(k)}$ and $C^{(k)}$ as:

$$\begin{aligned} [ER^P C]^{(k)} &= \frac{[C^{(k-1)}]}{[C^{(k-1)}] + k_{pdeg} \frac{k_7+k_8}{k_8 k_6}} \frac{k_5}{k_8} [ERP^P] \\ h^{(k)} &= \frac{f_{C1}(E)}{k_{pdeg}} - [ER^P C]^{(k)} \\ C_1^{(k)} &= \frac{k_{10}}{k_9} \frac{1}{2} \left(-1 + \sqrt{1 + 4 \frac{k_9}{k_{10}} h^{(k)}(E,C)} \right) \\ C^{(k)} &= \frac{k_9}{k_{10}} \left(C_1^{(k)} \right)^2 - [ER^P C]^{(k)} \end{aligned}$$

A2: If $C^{(k)} - C^{(k-1)} > \varepsilon_{toll}$, repeat from A1.

The previous procedure provides $[ER^P C]$, C_1 and C nullclines as a function of E.

An alternative procedure, which doesn't require the implementation of the iterative algorithm, is designed as follows:

- Compute $h(E) = \lim_{C\to+\infty} h(E,C)$, namely:

$$h(E) = \frac{f_{C1}(E)}{k_{pdeg}} - \frac{k_5}{k_8}[ERP^P]$$

- Compute C_1-nullcline as a function of E from equation (41), namely:

$$C_1 = \frac{k_{10}}{k_9}\frac{1}{2}\left(-1+\sqrt{1+4\frac{k_9}{k_{10}}h(E)}\right)$$

- Compute C-nullcline as a function of E from equation (40) with $C \to +\infty$, namely:

$$C = \frac{k_9}{k_{10}}C_1^2 - \frac{k_{pdeg}}{k_{10}}\frac{k_5}{k_8}[ERP^P]$$

- Compute $[ER^PC]$-nullcline from equation (39), namely:

$$[ER^PC] = \frac{C}{C + k_{pdeg}\frac{k_7+k_8}{k_8k_6}}\frac{k_5}{k_8}[ERP^P]$$

3.4.4. ER^P-nullcline as a function of E.

It follows from equations (32) and (35) that

$$\frac{k_5}{k_8}[ERP^P] - \frac{k_{pdeg}}{k_8}[ER^P] = \frac{k_6}{k_7+k_8}[ER^P][C]$$

Solving the above equation for $[ER^P]$ we obtain $[ER^P]$-nullcline as a function of E, namely:

$$[ER^P] = \frac{\frac{k_5}{k_8}[ERP^P]}{\frac{k_6}{k_7+k_8}[C] + \frac{k_{pdeg}}{k_8}} \qquad (42)$$

3.4.5. Total amount of SigE as a function of E.

Recalling the definition of E_T, the static relationship among E and E_T can finally be computed.

4. Including RseA dynamics within the model

So far we have assumed that RseA concentration is constant and equal to R_T. We now remove such an assumption, and consider RseA basal production and degradation and ClpC1P2-mediated degradation of phosphorylated RseA. To this aim, the following chemical reactions need to be taken into account:

◇ RseA basal production:
$\emptyset \xrightarrow{v_R} R$

◇ RseA basal degradation:
$\emptyset \xleftarrow{\delta_R} R$

◇ RseA binding to SigE: complex formation and dissociation:
$E + R \underset{k_4}{\overset{k_3}{\rightleftharpoons}} E|R$

◇ ClpC1P2-mediated degradation of phosphorylated RseA:
$E|R^P|C1|P2 \xrightarrow{k_8} E + C1|P2$

From the previous set of chemical reactions, the following differential equation describing dynamic evolution of RseA concentration can be readily obtained:

$$\frac{dR}{dt} = v_R - k_3ER + k_4[ER] - \delta_R R \tag{43}$$

The equilibrium condition $\frac{dR}{dt} = 0$ yields

$$v_R - k_3ER + k_4[ER] - \delta_R R = 0$$

By combining the previous equation with Eqn. (24) we get

$$\begin{aligned} R &= \frac{v_R}{\delta_R} - \frac{k_5}{\delta_R}[ERP^P] \\ &= R_{max}\left(1 - \frac{1}{R_{max}}\frac{k_5}{\delta_R}[ERP^P]\right) \end{aligned}$$

where we have set $R_{max} = \frac{v_R}{\delta_R}$, and hence

$$\frac{1}{R} = \frac{\delta_R}{R_{max}\delta_R - k_5[ERP^P]} \tag{44}$$

Now, rewrite equation (30) as

$$[ERP^P] = \frac{\overbrace{k_1 k_{ap}^{Pk} P_T}^{\alpha_n}[ER]}{\underbrace{k_1\left(k_5 + k_{ap}^{Pk}\right)}_{\alpha_d}[ER] + \underbrace{\left(k_{ap}^{Pk} + k_{ad}^{Pk}\right)(k_2 + k_5)}_{\beta_d}} = \frac{\alpha_n[ER]}{\alpha_d[ER] + \beta_d} \tag{45}$$

Plugging equation (45) into equation (44), we obtain

$$\frac{1}{R} = \frac{\delta_R\alpha_d[ER] + \delta_R\beta_d}{(R_{max}\delta_R\alpha_d - k_5\alpha_n)[ER] + R_{max}\delta_R\beta_d} \tag{46}$$

Rewrite equation (31) (recalling that now R is not constant) as

$$E = \frac{k_5}{k_3R}[ERP^P] + \frac{k_4}{k_3R}[ER] \tag{47}$$

Upon substituting $1/R$ with equation (46) and $[ERP^P]$ with equation (45) in equation (47), tedious computations lead to the following quadratic equation in the unknown $[ER]$:

$$\underbrace{\frac{1}{R_{max}}\frac{k_4}{k_3}\alpha_d}_{\mathcal{A}}[ER]^2 + \underbrace{\left\{\frac{1}{R_{max}}\frac{k_5}{k_3}\alpha_n + \frac{1}{R_{max}}\frac{k_4}{k_3}\beta_d - \left(\alpha_d - \frac{1}{\delta_R R_{max}}k_5\alpha_n\right)E\right\}}_{\mathcal{B}(E)}[ER]\underbrace{-\beta_d E}_{\mathcal{C}(E)}$$

As in the case with constant RseA, Descartes' rule of signs ensures that only one of the two solutions is positive, and hence the explicit solution for $[ER]$ is given by:

$$[ER] = \frac{-\mathcal{B}(E) + \sqrt{\mathcal{B}^2(E) - 4\cdot\mathcal{A}\cdot\mathcal{C}(E)}}{2\cdot\mathcal{A}}$$

Notice that when $R_{max} = R_T$ and $\delta_R \to +\infty$, the above quadratic equation in $[ER]$ reduces to the analogous quadratic equation of the case with constant RseA ($R \equiv R_T$).

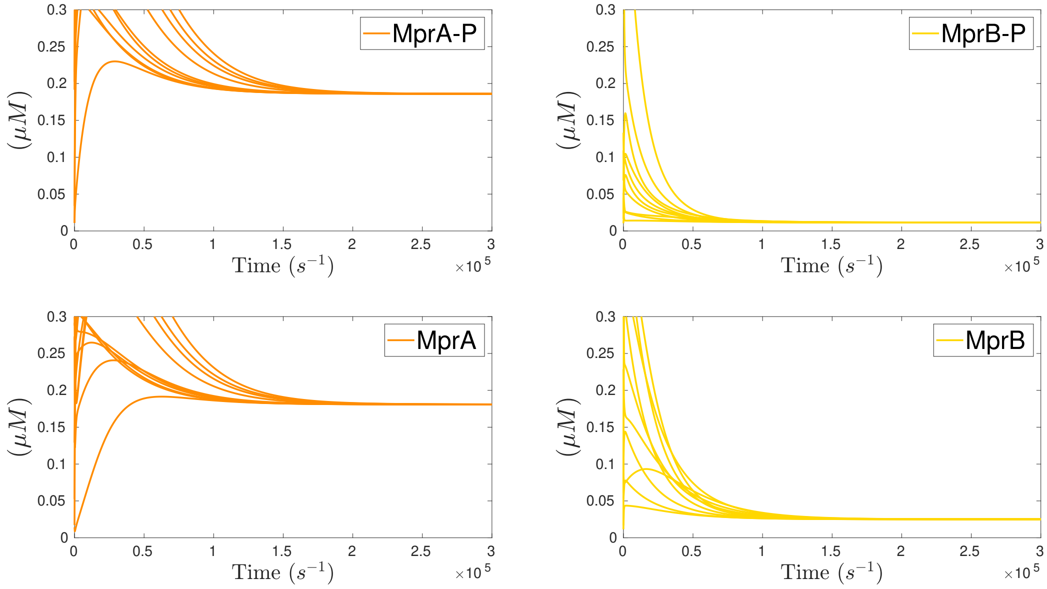

Figure 2: Simulation of the mathematical model obtained under the assumption of constant RseA concentration: temporal evolution of state variables A_P (upper left panel), B_P (upper right panel), A (lower left panel), B (lower right panel).

5. Model simulation

Figures 2-5 show dynamic evolution of model (1)-(14) obtained under the assumption of constant RseA concentration. Figures 6-9 report state variables evolution for the model described by differential equations (1)-(14) upon substitution of R_T with R, together with differential equation (43) (namely, the model describing SigE network after remove of constant RseA assumption). Parameters values are the same for both models and are reported in Additional file 2 – Table I.

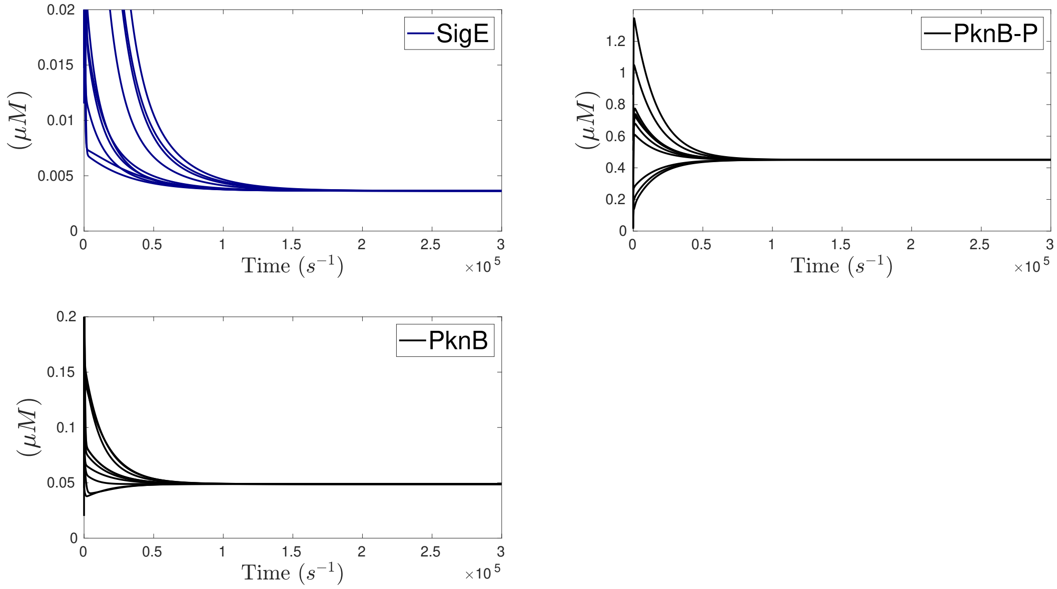

Figure 3: Simulation of the mathematical model obtained under the assumption of constant RseA concentration: temporal evolution of state variables E (upper left panel), P^P (upper right panel), P (lower left panel).

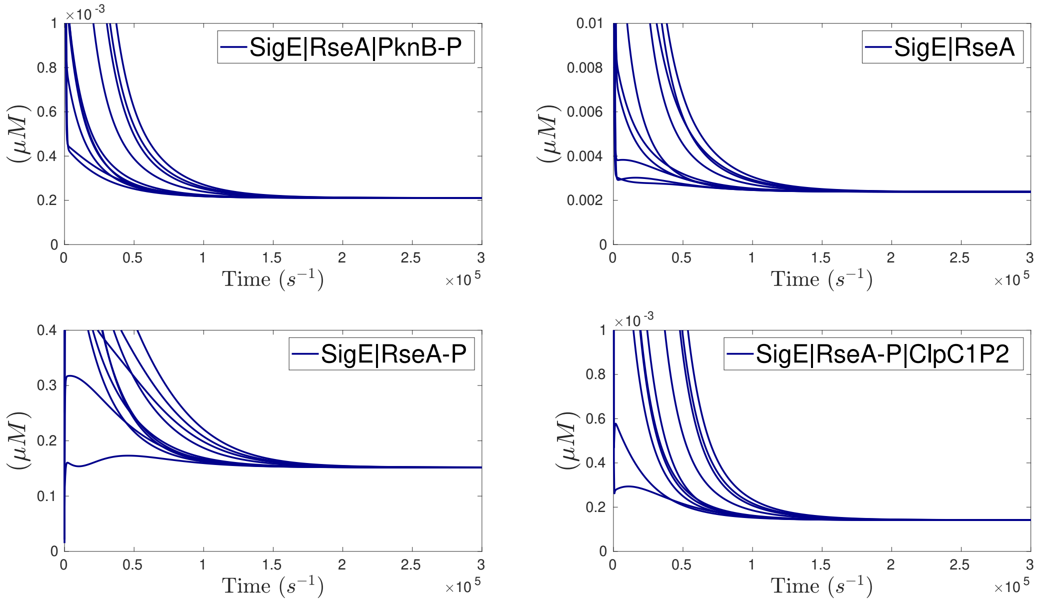

Figure 4: Simulation of the mathematical model obtained under the assumption of constant RseA concentration: temporal evolution of state variables $[ERP^P]$ (upper left panel), $[ER]$ (upper right panel), $[ER^P]$ (lower left panel), $[ER^PC]$ (lower right panel).

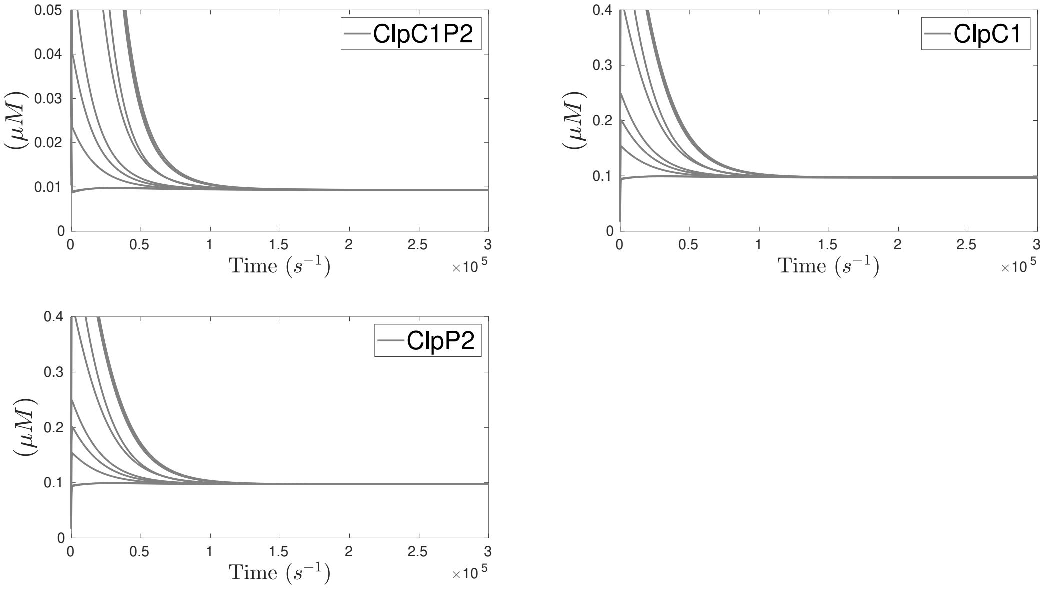

Figure 5: Simulation of the mathematical model obtained under the assumption of constant RseA concentration: temporal evolution of state variables C (upper left panel), C_1 (upper right panel), P_2 (lower left panel).

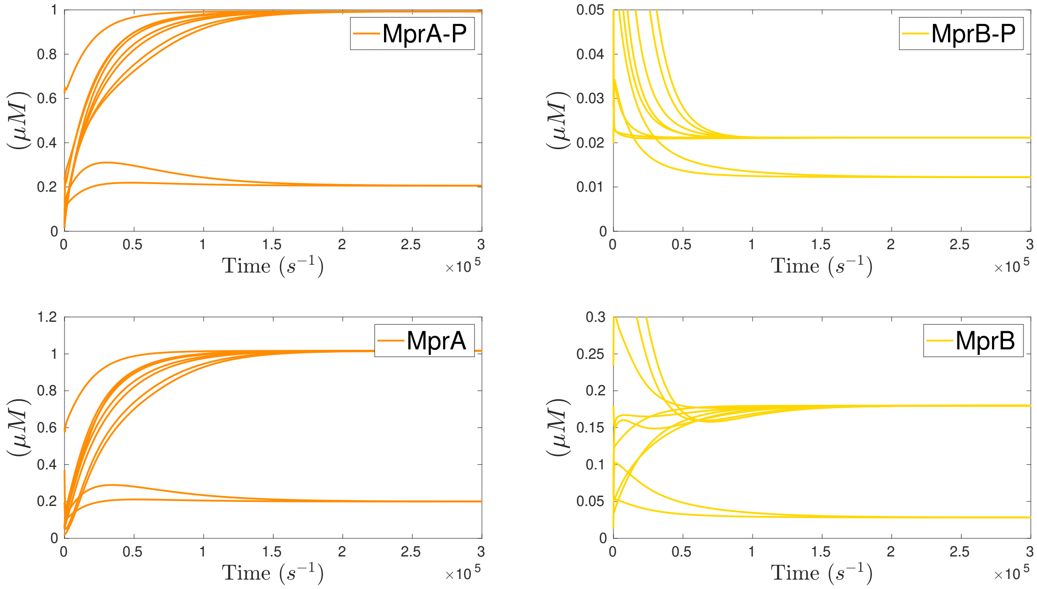

Figure 6: Simulation of the mathematical model obtained under the assumption of non-constant RseA concentration: temporal evolution of state variables A_P (upper left panel), B_P (upper right panel), A (lower left panel), B (lower right panel).

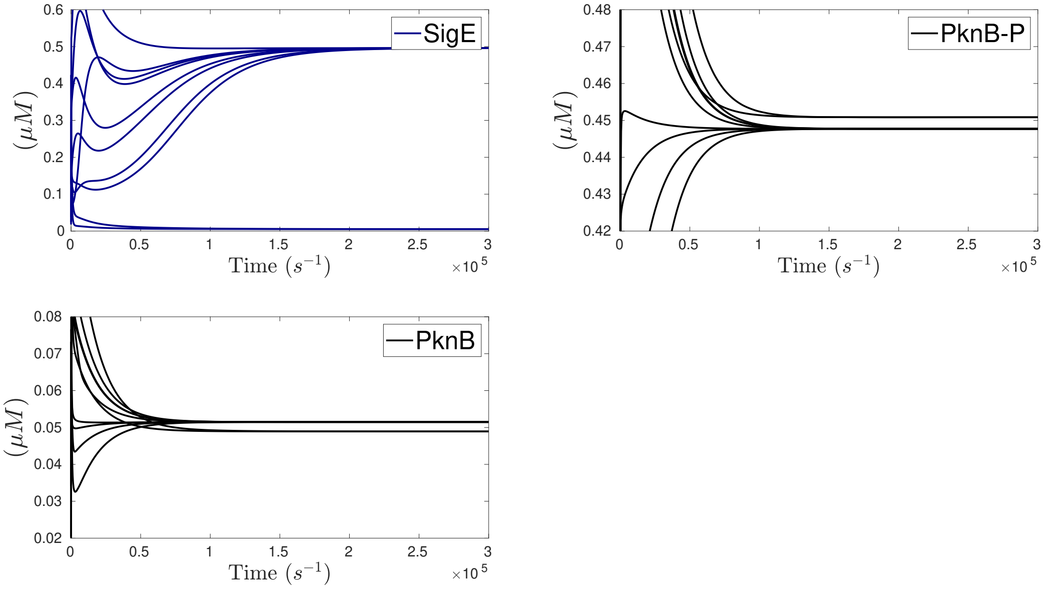

Figure 7: Simulation of the mathematical model obtained under the assumption of non-constant RseA concentration: temporal evolution of state variables E (upper left panel), P^P (upper right panel), P (lower left panel).

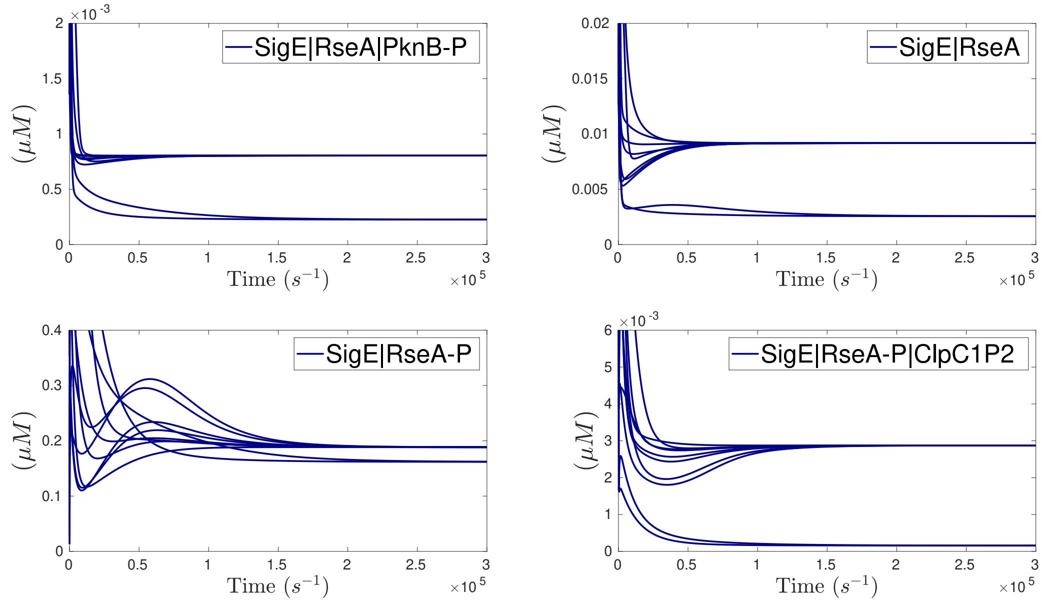

Figure 8: Simulation of the mathematical model obtained under the assumption of non-constant RseA concentration: temporal evolution of state variables $[ERP^P]$ (upper left panel), $[ER]$ (upper right panel), $[ER^P]$ (lower left panel), $[ER^PC]$ (lower right panel).

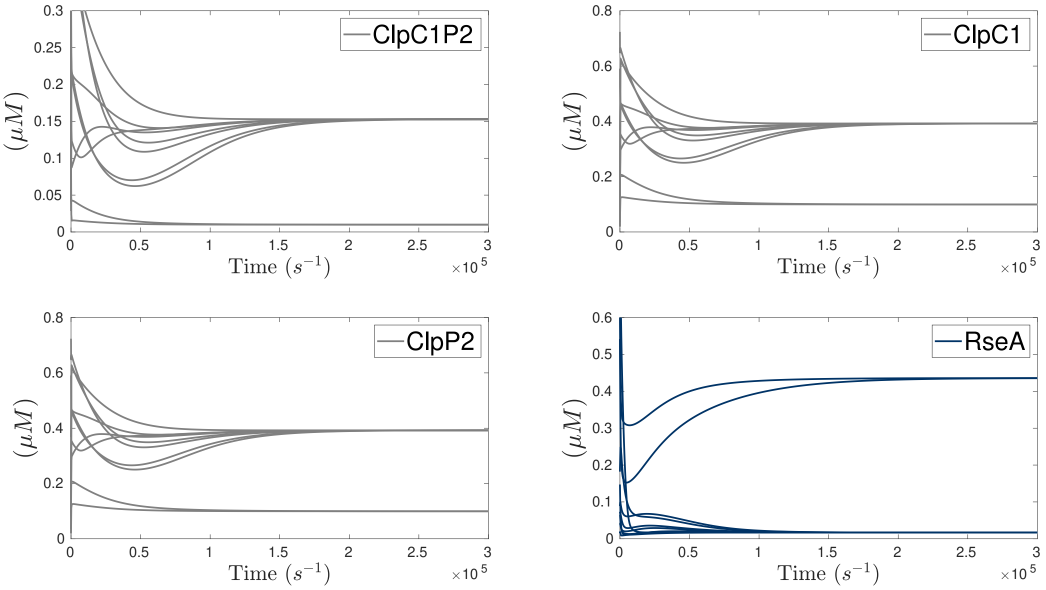

Figure 9: Simulation of the mathematical model obtained under the assumption of non-constant RseA concentration: temporal evolution of state variables C (upper left panel), C_1 (upper right panel), P_2 (lower left panel), R (lower right panel).

References

[1] A. Tiwari, G. Balazsi, M.L. Gennaro, and O.A. Igoshin. The interplay of multiple feedback loops with post-translational kinetics results in bistability of mycobacterial stress response. *Physical Biology*, 7(3), 2010.
