# Supplementary material for: Mathematical modelling of SigE regulatory network reveals new insights into bistability of mycobacterial stress response
Source: BMC Bioinformatics. 2021 Nov 19;22:558. doi: 10.1186/s12859-021-04372-5 (PMC8605609; doi:10.1186/s12859-021-04372-5)
Supplement: Supplementary file 2 — Additional file 2 - Table I. This table contains numerical values of model parameters. [file 12859_2021_4372_MOESM2_ESM.pdf]

Parameter	Value	Description
k_{pdeg}	$6 \cdot 10^{-5}\,s^{-1}$	Proteins degradation rate constant
k_{ad}	$0.001\,s^{-1}$	*MprB-P* dephosphorylation catalytic rate constant
k_{ap}	$0.00132\,s^{-1}$	*MprB* autophosphorylation rate constant
k_t	$0.5\,s^{-1}$	*MprB-P–MprA* phosphotransfer rate constant
k_p	$0.05\,s^{-1}$	*MprA-P* dephosphorylation by *MprB* rate constant
f_1	10	Amplification gain for promoter P^1_{AB}
f_2	10	Amplification gain for promoter P^2_{AB}
f_3	10	Amplification gain for promoter P_E
β_1	$6 \cdot 10^{-6}\,\mu M s^{-1}$	Basal transcription rate for promoter P^1_{AB}
β_2	$9 \cdot 10^{-6}\,\mu M s^{-1}$	Basal transcription rate for promoter P^2_{AB}
β_3	$6 \cdot 10^{-6}\,\mu M s^{-1}$	Basal transcription rate for promoter P_E
K_1	$0.5\,\mu M^2$	Dissociation constant for 2*MprA-P*–DNA binding
K_2	$0.08\,\mu M$	Dissociation constant for *SigE*–DNA binding
R_T	$0.6\,\mu M$	Total concentration of anti-sigma factor *RseA*
K_D	$3 \cdot 10^{-4}\,\mu M$	Equilibrium dissociation constant for *SigE–RseA* complex
K_T	$50\,\mu M$	Michaelis-Menten constant for *MprA–MprB-P* phosphotransfer
K_P	$50\,\mu M$	Michaelis-Menten constant for *MprA-P* dephosphorylation by *MprB*
λ	0.1	Ratio of *MprB* and *MprA* total concentrations
k_{exp}	$0.001\,s^{-1}$	*MprA* exogenous phosphorylation rate constant
k_{exd}	$0.001\,s^{-1}$	*MprA-P* exogenous dephopshorylation rate constant
k^{Pk}_{ad}	$0.001\,s^{-1}$	*PknB-P* dephosphorylation rate constant
k^{Pk}_{ap}	$0.01\,s^{-1}$	*PknB* phosphorylation rate constant
k_1	$0.01\,s^{-1}/\mu M$	Association rate constant for *SigE–RseA–PknB-P* complex
k_2	$0.001\,s^{-1}$	Dissociation rate constant for *SigE–RseA–PknB-P* complex
k_3	$0.006\,s^{-1}/\mu M$	Association rate constant for *SigE–RseA* complex
k_4	$0.001\,s^{-1}$	Dissociation rate constant for *SigE–RseA* complex
k_5	$0.05\,s^{-1}$	Rate constant for *PknB*-dependent phosphorylation of *SigE–RseA* complex
k_6	$0.002\,s^{-1}/\mu M$	Association rate constant for *SigE–RseA-P–C1–P2* complex
k_7	$0.01\,s^{-1}$	Dissociation rate constant for *SigE–RseA-P–C1–P2* complex
k_8	$0.01\,s^{-1}$	Rate constant for *ClpC1P2*-dependent degradation of *SigE–RseA-P* complex
k_9	$0.01\,s^{-1}/\mu M$	Association rate constant for *C1–P2* complex
k_{10}	$0.01\,s^{-1}$	Dissociation rate constant for *C1–P2* complex
β_{C1}	$6 \cdot 10^{-6}\,\mu M s^{-1}$	Basal transcription rate for promoter P_{C1}
f_{C1}	10	Amplification gain for promoter P_{C1}
K_{C1}	$0.5\,\mu M$	Dissociation constant for *ClgR*–DNA binding
β_{P2}	$6 \cdot 10^{-6}\,\mu M s^{-1}$	Basal transcription rate for promoter P_{P2}
f_{P2}	10	Amplification gain for promoter P_{P2}
K_{P2}	$0.5\,\mu M$	Dissociation constant for *ClgR*–DNA binding
P_T	$0.5\,\mu M$	Total concentration of *PknB*
δ_R	$7 \cdot 10^{-5}\,s^{-1}$	*RseA* degradation rate constant

TABLE I: **List of parameters values used in the model.** Numerical values for parameters above the horizontal line have been borrowed from [1], Table S3. Gray shaded rows indicate parameters whose numerical value differ from the value reported in the referred Table S3 of [1]: R_T is slightly varied but still within the bistability region shown in Figure S2 of [1]; k_{exd} equals k_{exp} to ensure perfect balance between exogenous phosphorylation and dephosphorylation fluxes (this choice is consistent with Figure 5 in [1] whose bifurcation diagrams have been obtained with both k_{exd} and k_{exp} set to zero). For parameters below the horizontal line, since estimate based on experimental data are not available, when analogous chemical reactions are present in [1], then corresponding parameters take similar values (see, e.g., basal transcription rate and amplification gain for promoters P_{C1} and P_{P2}). When this was not applicable (i.e., a similar chemical reaction is not considered in [1]), the order of magnitude is 10^{-3} or 10^{-2} like $\frac{k_p}{K_P}$ and $\frac{k_t}{K_T}$ in [1], respectively.

References

[1] A. Tiwari, G. Balazsi, M.L. Gennaro, and O.A. Igoshin. The interplay of multiple feedback loops with post-translational kinetics results in bistability of mycobacterial stress response. *Physical Biology*, 7(3), 2010.
